# Magnetic Reprogramming of Macrophages Stimulates Phagocytosis of Breast Cancer Cells via a TRPC1‐STING Inflammatory Axis

**DOI:** 10.1002/smmd.70038

**Published:** 2026-06-04

**Authors:** Viresh Krishnan Sukumar, Yee Kit Tai, Jan Nikolas Iversen, Olivia Yeo, Anisha Praiselin Paul, Kwan Yu Wu, Lina Hsiu Kim Lim, Alfredo Franco‐Obregón

**Affiliations:** ^1^ NUS Centre for Cancer Research Yong Loo Lin School of Medicine National University of Singapore Singapore; ^2^ BICEPS Lab (Biolonic Currents Electromagnetic Pulsing Systems) National University of Singapore Singapore; ^3^ Department of Surgery Yong Loo Lin School of Medicine National University of Singapore Singapore; ^4^ Institute for Health Innovation & Technology (iHealthtech) National University of Singapore Singapore; ^5^ School of Life Sciences and Chemical Technology Ngee Ann Polytechnic Singapore; ^6^ Department of Physiology Yong Loo Lin School of Medicine National University of Singapore Singapore; ^7^ Immunology Translational Research Program Department of Microbiology and Immunology Yong Loo Lin School of Medicine National University of Singapore (NUS) Singapore; ^8^ Immunology Programme Life Sciences Institute National University of Singapore Singapore; ^9^ Competence Center for Applied Biotechnology and Molecular Medicine University of Zurich Zurich Switzerland

**Keywords:** adaptive antitumor immunity, cyclic GMP‐AMP synthase (cGAS), immunotherapy, interferons, NF‐κB, pulsed electromagnetic field (PEMF), tumor microenvironment (TME), tumor‐associated macrophages (TAMs)

## Abstract

The reprogramming of tumor‐associated macrophages (TAMs) from a pro‐tumoral M2 to an anti‐tumoral M1 phenotype is an attractive therapeutic strategy whose clinical translation is undermined by the systemic toxicity of currently available pharmacological approaches. Here, we demonstrate that non‐invasive and localizable pulsed electromagnetic fields (PEMFs) induce macrophage reprogramming downstream of transient receptor potential canonical 1 (TRPC1) channel activation. Brief (10 min) PEMF exposure polarized macrophages toward an M1 phenotype by activating Stimulator of Interferon Genes (STING)‐dependent NF‐κB inflammatory pathways that were abolished by TRPC1 knockdown or inhibition. PEMF exposure directly enhanced the immunogenicity of breast cancer cells and modified macrophage‐cancer crosstalk to promote M1 macrophage polarization and the attraction of STING‐activated macrophages to the cancer cells. In co‐cultures, PEMF exposure stimulated macrophage‐mediated phagocytosis of cancer cells in a STING‐ and TRPC1‐dependent manner. In spheroids, PEMFs induced the reprogramming of TAMs to an M1 status and selectively enhanced infiltration of M1 macrophages, resulting in STING‐mediated phagocytosis of cancer cells. In mice, 2 weeks of twice‐weekly PEMF exposure resorbed engrafted tumors and selectively eliminated cancer cells within tumors while promoting immune cell recruitment. PEMFs offer a non‐invasive manner to locally reprogram TAMs within the tumor microenvironment to preferentially eliminate cancer cells.

## Highlights

- PEMFs reprogram macrophages toward the M1 state.
- PEMFs activate the STING pathway in a TRPC1‐dependent manner.
- PEMFs enhance cancer immunogenicity.
- PEMFs modulate macrophage‐cancer cell paracrine crosstalk.
- PEMFs enhance anti‐cancer immunity by promoting macrophage‐mediated cancer‐specific phagocytosis in vitro, ex vivo and in vivo.

## Graphical abstract

Magnetic fields modulate macrophage‐cancer crosstalk through the STING‐TRPC1 axis. Exposure reprograms macrophages toward the anti‐cancer phenotype. Exposing cancer cells modulates cancer secretome to promote polarization and selective recruitment of M1‐tumoricidal macrophages, enhancing cancer immunogenicity. Collectively, magnetic exposure enhances cancer phagocytosis and clearance in 2D and 3D spheroid co‐cultures; and in tumor‐bearing mice.

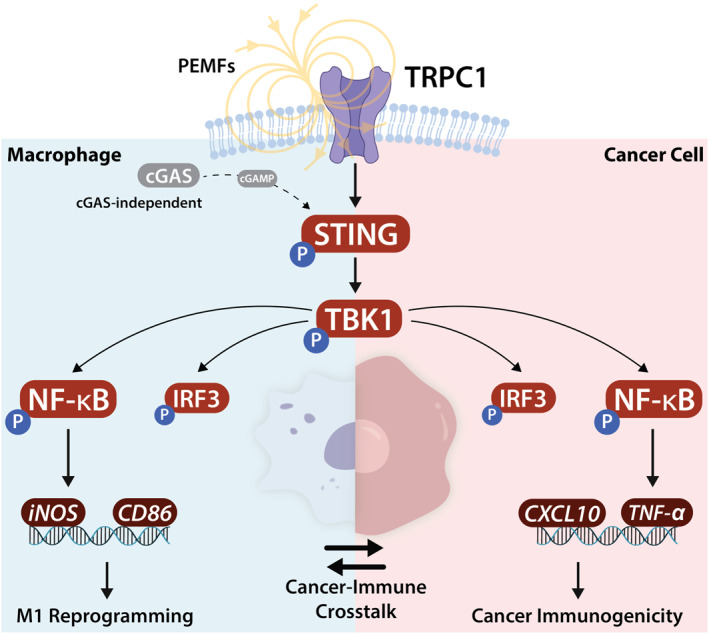

## 1 Introduction

Macrophages exhibit remarkable functional plasticity that enables them to act either as sentinels to detect and eliminate pathogens or as facilitators of tissue remodeling and repair [1]. This phenotypic duality is endowed by their capacity to metabolically polarize into distinct functional states in response to environmental cues. The classical M1 phenotype is promoted by physical interaction with microbial components, such as bacterial lipopolysaccharides (LPS) or viral DNA, mediated by pattern recognition receptors (PRRs), including Toll‐like receptor 4 (TLR4) and cGAS‐STING (cyclic GMP‐AMP synthase‐Stimulator of Interferon Genes) [2]. PRR engagement then triggers canonical NF‐κB (Nuclear Factor Kappa B) (p65) and STAT1 (Signal Transducer and Activator of Transcription 1) signaling, driving the expression of proinflammatory mediators, including iNOS (inducible Nitric Oxide Synthase), CD86 (Cluster of Differentiation 86), TNF‐α (Tumor Necrosis Factor Alpha), IL‐6 (Interleukin 6), and IL‐12 [3, 4], as well as the anti‐viral cytokines, IFN‐α (Interferon alpha) and IFN‐β [5]. In vitro, M1 polarization can be recapitulated by simultaneous exposure of macrophages to LPS and IFN‐γ, thereby activating NF‐κB and STAT1 [6]. The STAT1 and NF‐κB signaling pathways are thus key therapeutic targets for the modulation of macrophage inflammatory status in the potential management of cancer.

On the other hand, the M2, or alternatively activated, status is promoted by macrophage interaction with anti‐inflammatory cytokines such as IL‐4 and IL‐10, parasites (e.g., helminths), or immunomodulatory drugs. M2 polarized macrophages are characterized by the expression of IL‐10, TGF‐β (Transforming Growth Factor Beta), PPAR‐γ (Peroxisome Proliferator‐Activated Receptor gamma), Arginase, and CD206 [3]. M2‐polarized macrophages are commonly involved in tissue repair, wound healing and the resolution of inflammation.

Tumor‐associated macrophages (TAMs) are key regulators of the tumor microenvironment (TME). M2‐like TAMs typically predominate in the TME, where they facilitate tumor growth by secreting pro‐tumorigenic factors and by suppressing anti‐tumoral immunity [6]. By contrast, M1‐polarized TAMs exert anti‐tumoral effects by inducing apoptosis, promoting phagocytosis, and orchestrating an anti‐tumor immune response by recruiting CD4^+^ Th1 cells, cytotoxic CD8^+^ T cells, and NK cells into the TME [6, 7]. Underscoring the permissive role that M2‐like TAMs play in cancer progression, Nimesulide‐induced TAM depletion led to reduced tumor size in a murine lung cancer model [7, 8]. Similarly, PRR‐induced reprogramming of TAMs into an M1‐like state also regressed tumor growth in mice [7]. Accordingly, an elevated M1/M2 ratio correlates with improved patient outcomes and slower disease progression [9]. As TAMs comprise up to 50% of the total tumor mass, they are a promising therapeutic target for cancer management [10] and developing strategies to efficiently modulate macrophage polarization toward an M1‐status should reshape the TME toward an anti‐cancer status.

One emerging approach has been the use of PRR agonists to reprogram TAMs toward an M1‐like status. PRR agonists promote anti‐tumor immunity by stimulating the production of proinflammatory cytokines and cytotoxic immune responses [11]. Despite the initial successes of PRR agonists in preclinical models, they have faced significant clinical challenges in human trials due to off‐target systemic inflammatory effects and poor tumor penetrance [12]. Thus, an urgent need exists for the development of non‐drug‐based therapeutic modalities that are capable of reprogramming TAMs with minimal systemic toxicity.

The TRPC1 (Transient Receptor Potential Canonical 1) cation channel is a key regulator of macrophage polarization [13]. TRPC1‐mediated calcium influx is required for macrophage M1 polarization following IFNγ and LPS stimulation [13]. Accordingly, genetic silencing of TRPC1, or pharmacological inhibition of TRPC1‐mediated calcium permeation, attenuates the expression of classical M1 markers [13]. TRPC1 has also been implicated in magnetoreception [14], whereby TRPC1‐mediated calcium entry in response to pulsed electromagnetic field (PEMF) exposure is associated with activation of the PGC‐1α (peroxisome proliferator‐activated receptor gamma coactivator 1‐alpha) transcriptional program, thereby producing mitohormetic responses to oxidative stress [15]. TRPC1 overexpression has also been implicated in cancer progression [16], in which the developmental consequences of PGC‐1α activation are context‐dependent [17]. The sum of these diverse pieces of evidence hence points to TRPC1 as a potential molecular target for clinical intervention in breast cancer.

STING is an intracellular PRR that is activated by a cyclic GMP‐AMP analogue. 2′3′‐cGAMP is generated by cyclic GMP‐AMP synthase (cGAS) upon detecting cytosolic DNA fragments of either prokaryotic (bacterial or mitochondrial) or eukaryotic origins. The binding of 2′3′‐cGAMP to STING triggers interferon production to mount an anti‐viral response [4, 18]. STING also mediates interferon‐independent anti‐tumor defenses. Interferon‐independent STING defenses arise from the reprogramming of TAMs toward an M1 phenotype that, in turn, enhances cytokine‐dependent tumor immunogenicity, immune cell recruitment, and upregulates antigen presentation [18]. As macrophage upregulation of PGC‐1α results in STING activation [19], PEMF‐mediated activation of PGC‐1α may serve as a means to induce an anti‐tumor M1 macrophage polarization in a spatially controlled and non‐invasive manner without the limitations imposed by blood‐borne delivery of pharmacological agents to the TME.

One PEMF strategy has previously demonstrated the capacity of selectively compromising breast cancer cell viability across in vitro, ex vivo, and in vivo models [20, 21]. This particular PEMF paradigm demonstrated a cytotoxic predilection for breast cancer cells compared to other collateral cell classes [20, 21, 22]. This method of magnetic stimulation exhibits a mechanistic dependence on TRPC1 expression for the elaboration of preferential inflammatory cytotoxicity of breast cancer [20, 21, 22] and for the preferential uptake of doxorubicin into breast cancer cells [22], as well as has undergone a Phase 1 clinical trial with no apparent adverse consequences (ClinicalTrials.gov ID: NCT06332508). Nonetheless, while these previous studies have demonstrated promising selective anti‐cancer effects in isolated breast cancer cell models, the nature of the effect of this PEMF paradigm over TAMs has remained unexplored. To address this critical clinical gap, this study investigated the effects of this PEMF paradigm on TAM polarization within the TME in both in vitro and in vivo model systems.

## 2 Results

### 2.1 PEMFs Induce the Expression of M1‐Associated Markers in Naïve and IL‐4‐Polarized Macrophages

**FIGURE 1:**
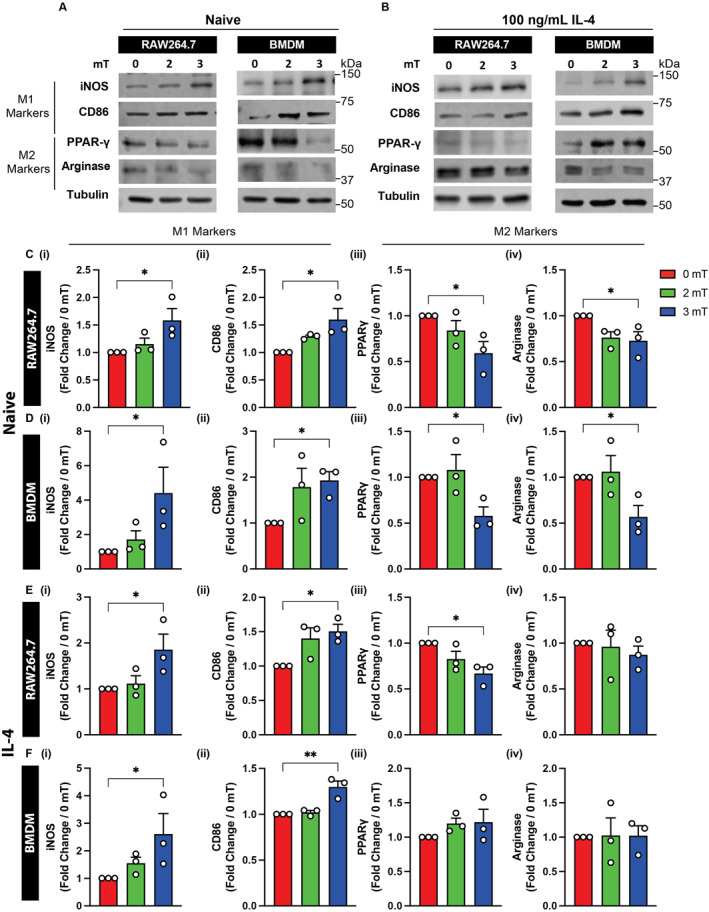
PEMF (3 mT, 10 min) induces changes in macrophage polarization markers. Representative western blots of (A) naïve and (B) IL‐4‐treated (100 ng/mL) RAW264.7 cells and bone marrow‐derived macrophages (BMDMs) for proteins as indicated. Quantitative analysis showing fold changes in (C) naïve RAW264.7, (D) naïve BMDMs, (E) IL‐4‐treated RAW264.7, and (F) IL‐4‐treated BMDMs for: (i) iNOS, (ii) CD86, (iii) PPAR‐*γ*, and (iv) Arginase. All protein levels were normalized to the loading control. All samples were normalized to the corresponding 0 mT control. Dots represent individual biological replicates. Data represent mean ± SEM (*n* = 3). Statistical significance was determined by one‐way ANOVA with Dunnett's post hoc test (*p ≤ 0.05 and **p ≤ 0.01).

Macrophage polarization plays a pivotal role in immune regulation, whereby the pro‐inflammatory M1 and anti‐inflammatory M2 phenotypes exert distinct functional and therapeutic effects. To evaluate the potential of PEMF exposure to modulate macrophage polarization, naïve murine RAW264.7 macrophages (Figure 1A) or bone marrow‐derived macrophages (BMDM) (Figure 1B) were exposed to varying amplitudes of PEMFs (0, 2, and 3 mT) for 10 min. Quantitative protein analysis was performed by Western blotting 24 h post‐exposure to assess polarization status. For both macrophage classes, exposure to 3 mT PEMFs (blue bars, Figure 1) induced the greatest expression of M1‐associated protein markers, specifically increasing the levels of NF‐κB‐regulated proteins [23] iNOS and CD86, which are characteristic of the M1 phenotype, with concurrent reductions in the M2 markers, PPAR‐γ and Arginase (Figure 1C,D).

To model the pro‐tumoral M2 phenotype of TAMs, RAW264.7 and BMDM macrophages were pretreated with 100 ng/mL IL‐4 for 24 h before PEMF exposure (Supporting Information S1: Figure S1A) [24]. Following 3 mT PEMF exposure (10 min), both macrophage classes exhibited a shift toward an M1‐like phenotype as evidenced by increased expression of iNOS (Figure 1Ei,ii) and CD86 (Figure 1Fi,ii). IL‐4 pretreated RAW264.7 macrophages also showed decreased expression of the M2 marker, PPAR‐*γ*, after PEMF exposure, while IL‐4‐treated BMDMs did not (Figure 1Eiii,iv,Fiii,iv). Additionally, at 48 h, both naïve and IL‐4‐polarized RAW264.7 macrophages demonstrated a persistent PEMF‐associated upregulation of iNOS and CD86 (M1 markers), coincident with a downregulation of PPAR‐γ (M2 marker) (Supporting Information S1: Figure S1B–D). However, M2 marker CD206 expression remained unchanged (Supporting Information S1: Figure S1E–F). This observation is consistent with the literature as CD206 expression may not necessarily reflect an accurate M2/M1 transition following specific stimuli, such as LPS treatment [25]. These findings demonstrate that 3 mT PEMF exposure can effectively polarize distinct macrophage classes toward a pro‐inflammatory M1 phenotype.

### 2.2 PEMF Activation of NF‐κB Is STING Dependent

**FIGURE 2:**
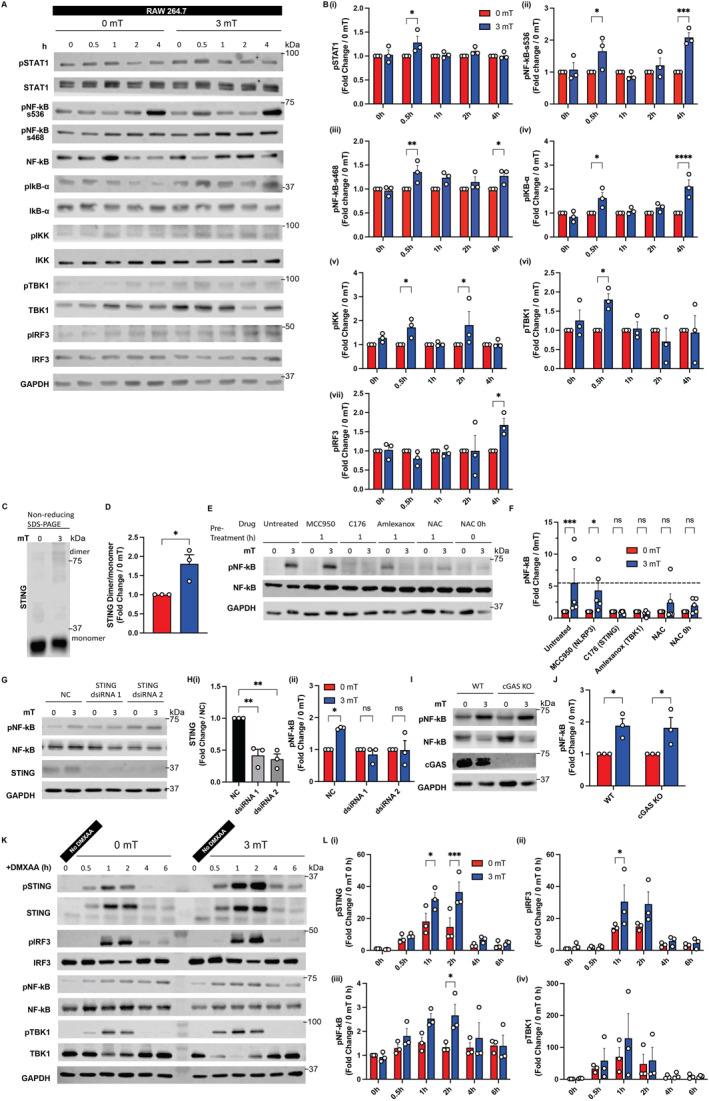
PEMF (3 mT, 10 min) activates the STING pathway in RAW264.7 macrophages. (A) Representative western blots and (B) fold change quantification of phosphorylated (i) STAT1, (ii) NF‐κB (s536), (iii) NF‐κB (s468), (iv) IκB‐α, (v) IKK, (vi) TBK1 and (vii) IRF3 in a time‐course study after PEMF exposure. (*n* = 3). (C) Representative western blot run on non‐reducing SDS‐PAGE and (D) fold change quantification of STING dimers normalized to STING monomer. (*n* = 3). (E) Representative western blots and (F) fold change quantification of phosphorylated NF‐κB 4 h after PEMF exposure in cells pretreated with inhibitors of NLRP3 (0.5 μM MCC950), STING (2 μM C176), TBK1 (100 μM Amlexanox), and ROS (5 mM N‐acetylcysteine, NAC) for 1 h (*n* = 5). (G) Representative western blots and (H) fold change quantification of (i) STING and (ii) phosphorylated NF‐κB 4 h post PEMF exposure. RAW264.7 macrophages were analyzed 24 h post‐transfection with non‐targeting dsiRNA (NC) or dsiRNA against STING (*n* = 3). (I) Representative western blots and (J) fold change quantification of phosphorylated NF‐κB 4 h post PEMF exposure in wildtype and cGAS knock‐out RAW264.7 cells (*n* = 3). (K) Representative western blots and (L) fold change quantification of phosphorylated (i) STING, (ii) IRF3, (iii) NF‐κB and (iv) TBK1 in a time‐course study after treatment with 20 μg/mL DMXAA and 10‐min PEMF (3 mT). PEMF exposure was administered immediately after DMXAA treatment (*n* = 3). All phosphoprotein levels were normalized to total protein, except for phosphorylated STING and phosphorylated NF‐κB, which were normalized to the loading control. Residual antibody binding to the phosphorylated form of NF‐κB hindered the reprobing of total NF‐κB. Phosphorylated NF‐κB was normalized to the loading control, with total NF‐κB assessed on a separate gel. All samples were normalized to their time/drug matched 0 mT control except in (L) where samples were normalized to 0 mT at 0 h. Dots represent individual biological replicates. Data represent mean ± SEM. Statistical significance was determined by Student's unpaired *t*‐test (D), one‐way (H (i)) or two‐way ANOVA (B, F, H (ii), J, L) with Šidák's post hoc test (*p ≤ 0.05, **p ≤ 0.01, ***p ≤ 0.001 and ****, *p* ≤ 0.0001).

STAT1 and NF‐κB (p65) are central transcriptional regulators of the M1 phenotype following STING activation [3]. STAT1 and NF‐κB showed distinct phosphorylation kinetics following PEMF exposure (3 mT). Time‐course analyses by Western blot revealed that PEMF exposure increased STAT1 phosphorylation at 0.5 h (Figure 2A,Bi). Concurrently, NF‐κB showed activation of phosphorylation at both Ser536 and Ser468 at 0.5 and 4 h (Figure 2Bii,iii). NF‐κB activation coincided with the simultaneous phosphorylation of its upstream regulators IκB (I Kappa B) and IKK (I Kappa B Kinase) (Figure 2B iv,v). Given that NF‐κB phosphorylation at Ser536 is more thoroughly characterized with STING activation [26], this phosphorylation site is subsequently used in downstream experiments. Early STING pathway activation was evidenced by TBK1 (TANK‐Binding Kinase 1) phosphorylation at 0.5 h, which preceded the phosphorylation of both NF‐κB and IRF3 (Interferon Regulatory Factor 3) (Figure 2Bvi). Notably, while NF‐κB showed biphasic activation at 0.5 and 4 h timepoints (Figure 2Bii,iii), IRF3 phosphorylation was exclusively observed at 4 h (Figure 2Bvii), suggesting temporally regulated branching of the STING‐TBK1 signaling axis. STING dimerization was also observed following PEMF exposure, further supporting the activation of the STING pathway (Figure 2C,D).

The specificity of PEMF‐induced STING‐TBK1 activation was confirmed through pharmacological inhibition of both STING and its downstream effector TBK1. While vehicle‐treated controls (untreated) showed robust NF‐κB phosphorylation (Figure 2E,F), STING inhibition (2 μM C176, 1 h pretreatment) completely abolished PEMF‐mediated activation. TBK1 inhibition (100 μM Amlexanox, 1 h pretreatment) [31] similarly prevented NF‐κB phosphorylation (Figure 2E,F). As a negative control, inhibition of the unrelated NLRP3 inflammasome pathway (0.5 μM, MCC950, 1 h pretreatment) [27] had no effect on PEMF‐induced NF‐κB activation. Consistent with findings that ROS modulate immune responses [28] and can be produced by PEMF exposure [15, 21, 22], administration of the antioxidant, N‐acetylcysteine (NAC) (5 mM), 1 h before, or immediately prior to, PEMF exposure, partially reduced PEMF‐mediated STING signaling. The PEMF‐mediated activation of the STING‐TBK1 axis was further corroborated using two independent dicer‐substrate silencing RNAs (dsiRNA) targeting the STING transcript (Figure 2G,Hi). STING‐silenced RAW264.7 cells exhibited attenuated levels of phosphorylated NF‐κB 4 h after PEMF exposure (Figure 2Hii) relative to cells transfected with non‐targeting RNA (negative control, NC).

Cyclic GMP‐AMP synthase (cGAS) is an enzyme positioned upstream of STING activation in the innate immune response. cGAS is activated upon its detection of cytosolic DNA, catalyzing the synthesis of cGAMP, which initiates downstream STING signaling [29]. To determine whether PEMF‐induced STING activation requires cGAS signaling, cGAS‐knockout (KO) RAW264.7 macrophages were exposed to PEMF treatment. cGAS deficiency failed to impair PEMF‐induced NF‐κB phosphorylation (Figure 2I,J), indicating that PEMFs activate STING through a cGAS‐independent mechanism. Next, a potential synergism between direct and PEMF‐mediated STING stimulation was investigated using the STING agonist, DMXAA [29]. DMXAA (5,6‐dimethylxanthenone‐4‐acetic acid) functions as a cGAMP‐mimetic that binds to the STING dimer's active site, triggering downstream signaling cascades. 20 μg/mL DMXAA treatment of RAW264.7 triggered robust STING‐TBK1‐IRF3‐NF‐κB phosphorylation at 1 and 2 h post‐treatment. Notably, 3 mT PEMF exposure significantly amplified this activation (Figure 2K,L), with the exception of STAT1 phosphorylation, which remained unaffected (Supporting Information S1: Figure S2A). To quantify this interaction, we normalized the DMXAA and PEMF co‐treated conditions to PEMFs alone and observed a synergistic effect specifically for STING and IRF3 phosphorylation (Supporting Information S1: Figure S2B–E). These findings define a cGAS‐independent, PEMF‐mediated, STING‐TBK1 signaling axis that acts as a potent enhancer of M1 reprogramming.

### 2.3 TRPC1 Contributes to PEMF‐Induced STING Activation

**FIGURE 3:**
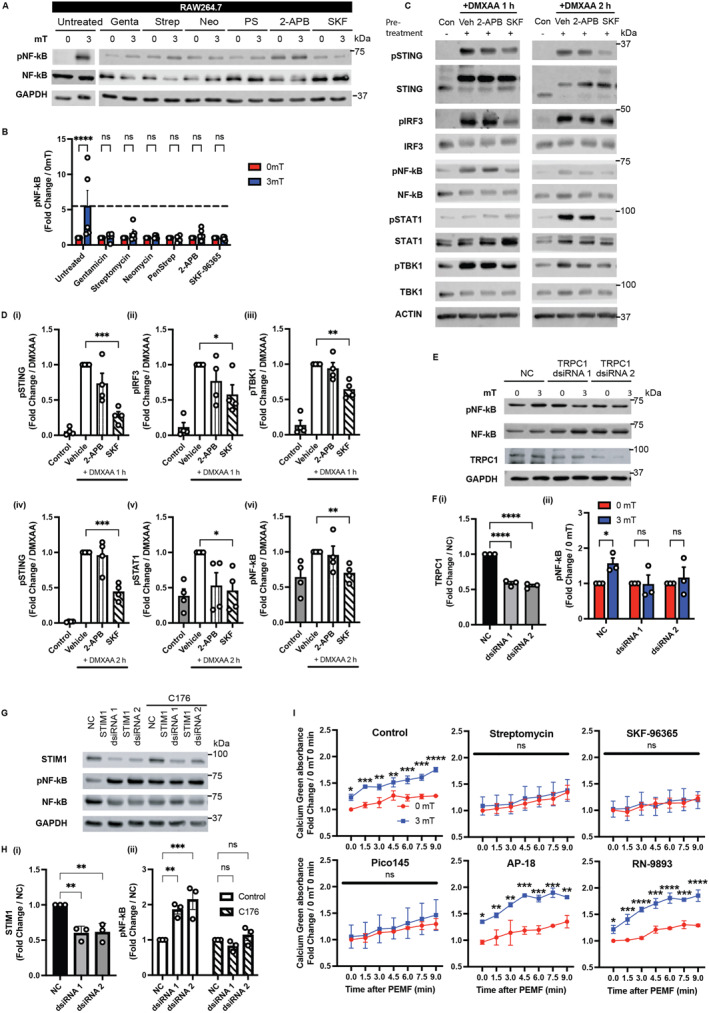
TRPC1 contributes to STING activation (A) Representative western blots and (B) quantification of phosphorylated NF‐κB 4 h after PEMF exposure in RAW264.7 cells pretreated for 1 h with aminoglycoside antibiotics (gentamicin, streptomycin, neomycin, penicillin‐streptomycin) or TRPC inhibitors (10 μM 2‐APB, 50 μM SKF‐96365). All samples were normalized to their drug‐matched 0 mT control (*n* = 5). (C) Representative western blots showing phosphorylated proteins following DMXAA treatment (20 μg/mL) in TRPC‐inhibited macrophages after 1 and 2 h post‐treatment. Bar charts in (D) show quantification of phosphorylated STING, IRF3, and TBK1 at 1 h (i–iii), and of STING, STAT1, and NF‐κB at 2 h (iv–vi). All samples were normalized to the vehicle control (*n* = 4). (E) Representative western blots of TRPC1‐silenced macrophages after PEMF exposure and (F) quantification of (i) TRPC1 and (ii) phosphorylated NF‐κB (*n* = 3). (G) Representative western blots of STIM1‐silenced macrophages in the presence of STING inhibitor C176, with (H) quantification of (i) STIM1 and (ii) phosphorylated NF‐κB. (I) Normalized Calcium Green absorbance comparing real‐time calcium influx in RAW264.7 cells immediately after PEMF exposure, highlighting the inhibitory effects of 15 min pre‐treatment with Streptomycin, SKF‐96365, Pico145, AP‐18 and RN‐9893 (*n* = 3). Phosphoprotein levels were normalized to their respective total protein levels, with the exceptions of phosphorylated STING and phosphorylated NF‐κB, which were normalized to GAPDH/β‐actin loading control. Dots represent individual biological replicates. Data represent mean ± SEM. Statistical significance was determined by one‐way (D, F (i), H (i)) or two‐way (B, F (ii), H (ii), I) ANOVA with Šidák's post hoc test (*p ≤ 0.05, **p ≤ 0.01, ***p ≤ 0.001 and ****, *p* ≤ 0.0001).

The transient receptor potential canonical 1 (TRPC1) cation channel has been convincingly implicated in magnetoreception [15, 21, 22, 30, 31] and is essential for M1 macrophage reprogramming [13]. Pharmacological antagonism of TRPC1 was used to elucidate its contribution to the PEMF‐mediated polarization of macrophages (Figure 3A,B). SKF‐96365 inhibits TRPC1 channel activation [32, 33, 34] by allosterically blocking its CaM/IP3R binding (CIRB) domain [35], whereas the aminoglycoside antibiotics (gentamicin, streptomycin, neomycin) sterically obstruct Ca^2+^ entry via open TRPC1 channels [36]. The aminoglycoside antibiotics, as well as the conventional tissue culture antibiotic (penicillin‐streptomycin, PS), applied only 1 h before PEMF exposure, suppressed NF‐κB phosphorylation. This result was recapitulated by acute pretreatment with two other TRPC channel antagonists, 10 μM 2‐APB and 50 μM SKF‐96365 ^34^ (Figure 3A,B). These findings support the role of TRPC1 in PEMF‐mediated M1 polarization of macrophages.

To investigate the necessity of TRPC1 in mediating STING‐NF‐κB signaling, RAW264.7 macrophages were treated with 20 μg/mL DMXAA, a STING agonist, which alone promoted the phosphorylation of STING, TBK1, IRF3, STAT1, and NF‐κB (Figure 3C,D). However, pretreatment with TRPC inhibitors for 1 h significantly attenuated DMXAA‐induced phosphorylation of these signaling components. SKF‐96365 produced strong suppressions, particularly of STING (1 and 2 h post‐treatment), IRF3 and TBK1 (1 h) (Figure 3C,Di–iv, Supporting Information S1: Figure S3), and STAT1 and NF‐κB (2 h) (Figure 3C,Dv–vi, Supporting Information S1: Figure S3). Consistent with the broader specificity of 2‐APB for numerous TRPC subclasses and other TRP channel family members [37], 2‐APB treatment resulted in a less pronounced inhibition profile [38]. The consistent suppression of these key phosphoproteins by TRPC inhibitors corroborates that TRPC1 channel activity is fundamentally required for STING‐NF‐κB pathway stimulation. This requirement was confirmed by the genetic silencing of TRPC1. Transient knockdown of TRPC1 using two independent dsiRNA attenuated PEMF‐induced NF‐κB activation compared to a non‐targeting control (NC) dsiRNA (Figure 3E,F). TRPC1's role in regulating intracellular calcium homeostasis depends on STIM1 [39], which also regulates STING function [40]. Transient silencing of STIM1 resulted in activation of NF‐κB, which was attenuated upon STING inhibition (C176) (Figure 3G,H), revealing opposing roles for TRPC1 and STIM1 in the STING pathway.

Finally, the role of TRPC1 in mediating calcium influx following PEMF exposure was examined using calcium green fluorimetry. In accordance with prior reports [15], PEMF exposure immediately increased intracellular calcium levels (Figure 3I). Inhibition of TRPC1 by pre‐treatment with Streptomycin, SKF‐96365, or Pico145 completely attenuated the PEMF‐induced calcium influx in RAW264.7 macrophages (Figure 3I). Apart from TRPC1, TRPV4 has been reported to regulate M1 macrophage polarization [41], while TRPA1 has been shown to promote NF‐κB‐dependent inflammation [42]. Inhibition of TRPV4 or TRPA1 did not affect PEMF‐induced calcium influx (Figure 3I). These data support the interpretation that TRPC1 mediates calcium influx and downstream signaling resulting from PEMF exposure.

### 2.4 PEMF Activation of TRPC1‐STING‐NF‐κB Signaling in Cancer Generates an Immunogenic Secretome Driving Macrophage Polarization

**FIGURE 4:**
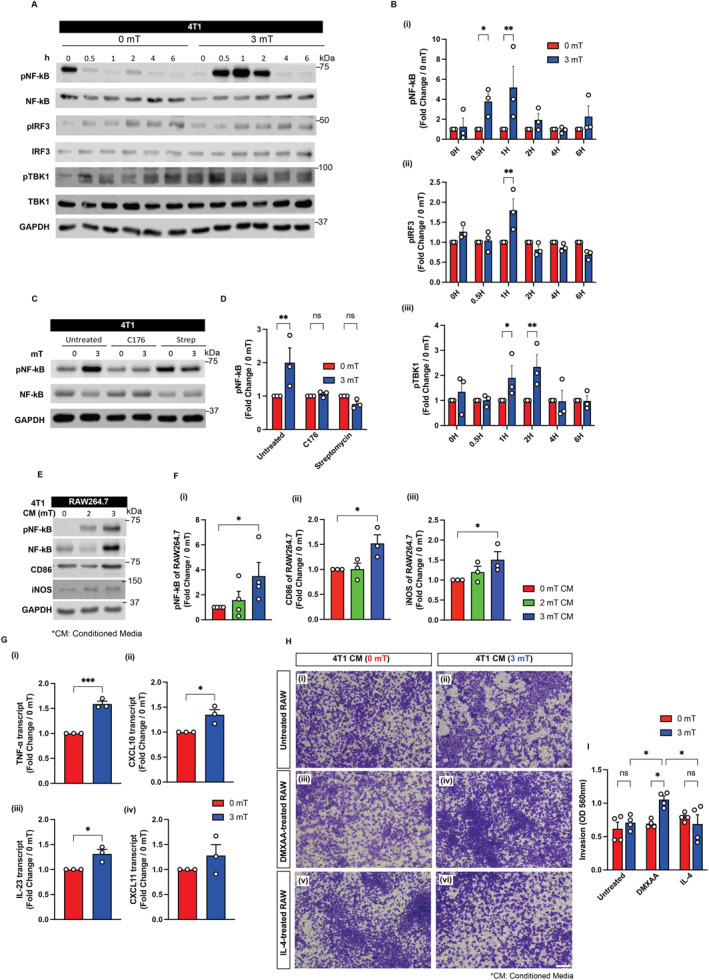
PEMF (3 mT, 10 min) activates STING pathway in 4T1. (A) Representative western blots and (B) fold change quantification of phosphorylated (i) NF‐κB, (ii) IRF3, and (iii) TBK1 in a time‐course study after PEMF exposure (*n* = 3). (C) Representative western blots and (D) fold change quantification of phosphorylated NF‐κB 1 h after PEMF‐exposure of 4T1 pre‐treated with STING (2 μM C176) or TRPC1 (streptomycin) inhibitors for 1 h (*n* = 3). (E) Representative western blots and (F) fold change quantification of (i) phosphorylated NF‐κB (ii) CD86 and (iii) iNOS in RAW264.7 macrophages after 24 h incubation in PEMF‐conditioned media from 4T1 (*n* = 4/3). Phosphoprotein levels were normalized to their respective total protein abundances, except for phosphorylated NF‐κB, which was normalized to the loading control. Residual antibody binding to the phosphorylated form of NF‐κB hindered the reprobing of total NF‐κB. Phosphorylated NF‐κB was normalized to the loading control, with total NF‐κB assessed on a separate gel. (G) qPCR analysis of (i) TNF‐α, (ii) CXCL10, (iii) IL‐23, (iv) CXCL11 transcripts normalized to β‐2M in 4T1 cells 3 h post PEMF exposure (*n* = 3 with 3 technical replicates each). (H) Representative images of invading untreated RAW264.7 macrophages in (i) 0 mT or (ii) 3 mT conditioned media (CM); (iii) 0 mT or (iv) 3 mT CM on DMXAA‐treated macrophages; (v) 0 mT or (vi) 3 mT CM on 100 ng/mL IL‐4‐treated macrophages. Scale bar = 100 μm. (I) Bar chart showing the corresponding quantifications. 4T1 cells were conditioned for 24 h to generate CM. Macrophages were incubated in CM for 48 h prior to imaging and analysis (*n* = 4 with 2 technical replicates each). All samples were normalized to their time/drug‐matched 0 mT control. Data represent the mean ± the standard error of the mean (SEM). Statistical analysis was performed using Student's unpaired *t*‐test (G), one‐way (F) or two‐way (B, D, I) ANOVA, followed by Šidák's multiple comparison post hoc test. Statistical significance is indicated by *, *p* ≤ 0.05; **, *p* ≤ 0.01, and ***p ≤ 0.001.

We previously showed that PEMF exposure polarized macrophages toward the pro‐inflammatory M1 phenotype. Identical PEMF exposure of 4T1 cancer cells also significantly enhanced the phosphorylation of TBK1 and downstream targets, NF‐κB and IRF3 (Figure 4A,Bi–iii) within 6 h. To determine whether this response depended on the same STING‐ and TRPC1‐dependent mechanism identified in macrophages, 4T1 cells were acutely pre‐treated with a STING inhibitor (C176; 2 μM) or TRPC1 open channel blocker (streptomycin) shortly (1 h) prior to PEMF exposure. Consistent with the results in RAW264.7 macrophages, both inhibitor classes attenuated PEMF‐induced NF‐κB phosphorylation (Figure 4C,D), demonstrating that PEMFs activate a conserved TRPC1‐STING‐TBK1‐NF‐κB pathway in both immune and cancer cells. The capacity of TRPC1, but not TRPV4 or TRPA1, to facilitate PEMF‐induced calcium influx was further confirmed in 4T1 cells (Supporting Information S1: Figure S4).

The cancer secretome is a key mediator of immunosuppression in the TME [43]. Accordingly, 4T1 breast cancer cell‐conditioned media has been shown to drive pro‐tumoral M2 macrophage polarization [44]. Conversely, STING activation in cancer cells enhances their immunogenicity, suggesting a potential countermeasure to immune suppression [18]. Given the context‐dependent nature of NF‐κB signaling in cancer, which can create either an immunostimulatory or immunosuppressive microenvironment depending on factors such as post‐translational protein modifications, cancer types, and the local immune milieu [45], we investigated whether PEMF‐induced NF‐κB activation in cancer cells could reprogram their secretome to favor an M1‐polarizing state in macrophages. To this end, RAW264.7 macrophages were treated with conditioned media from 4T1 cells exposed to PEMF (2 mT or 3 mT). Notably, media from PEMF‐treated 4T1 cells, particularly at 3 mT, significantly enhanced the phosphorylation of M1‐associated NF‐κB and upregulated the expressions of iNOS and CD86 in RAW264.7 cells within 24 h of treatment (Figure 4E,F). These results indicate that PEMF treatment alters cytokine expression in cancer cells, skewing macrophage polarity toward an M1‐like phenotype.

TNF‐α is a pro‐inflammatory cytokine that promotes M1 macrophage polarization [11], whereas the chemokines CXCL10 and CXCL11 serve as ligands for the CXCR3 receptor, which facilitates the recruitment of immune cells, including macrophages, into the TME [46]. Similarly, IL‐23, a member of the IL‐12 interleukin family, is known to stimulate the recruitment and activation of inflammatory immune cells into the TME [47]. Consequently, the enhanced expression of IL‐23, CXCL10, and CXCL11 in response to PEMF exposure may indicate a heightened inflammatory immune cell recruitment. Transcriptional analysis of NF‐κB‐regulated cytokines [45] in 4T1 cells 3 h post‐PEMF exposure demonstrated significant gene upregulations of *TNF‐α*, *CXCL10*, and *IL‐23* levels, but not *CXCL11* (Figure 4Gi–iv). Conditioned media (CM) from PEMF‐exposed 4T1 cells were next assayed for their ability to modulate macrophage chemotaxis. Given that tumor promoting TAMs are described to be M2‐like [3, 6], M2‐inducer IL‐4 was used to polarize macrophages to the M2 phenotype. While 4T1 PEMF‐CM did not significantly alter the migration of naïve macrophages (Figure 4Hi–ii), it increased the recruitment of DMXAA‐treated (M1‐like) macrophages (Figure 4Hiii–iv) but not IL‐4‐treated (M2‐like) macrophages (Figure 4Hv–vi)). This result aligns with prior reports of selective CXCL10‐mediated recruitment of M1 macrophages [46]. These findings demonstrate that PEMF exposure enhances cancer cell immunogenicity by stimulating the secretion of cytokines that drive M1 polarization and recruitment into the TME.

### 2.5 PEMF‐Induced TRPC1‐STING Axis Drives Macrophage Phagocytosis of 4T1 Cells

**FIGURE 5:**
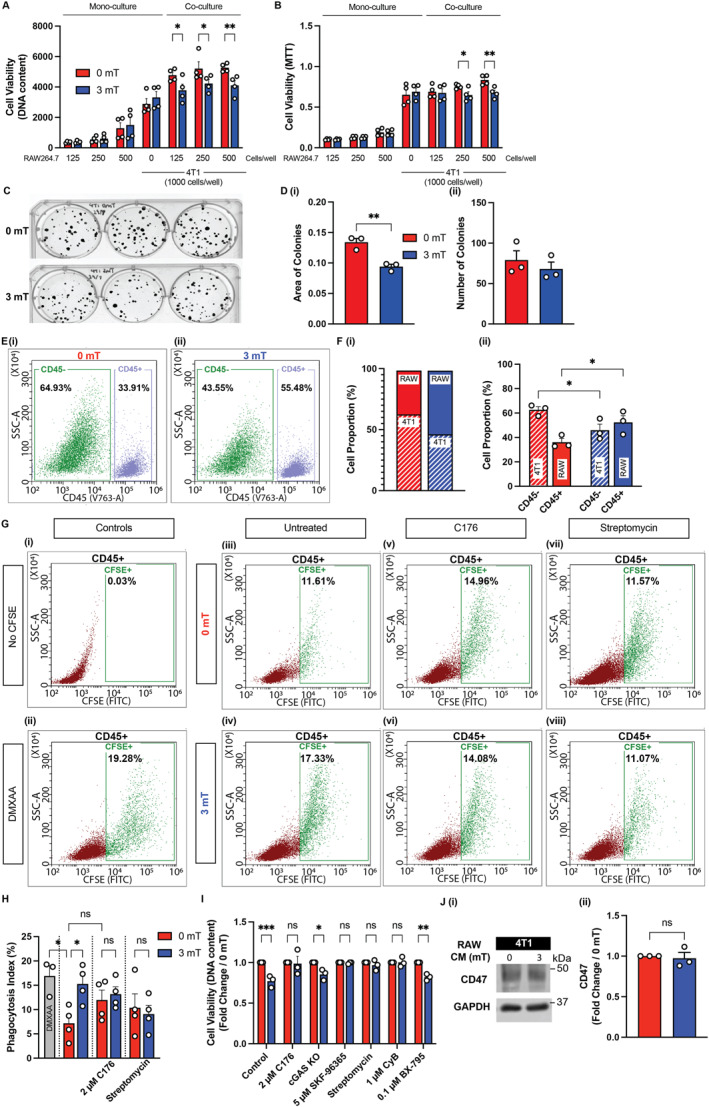
PEMF enhances macrophage‐mediated cancer cell clearance via STING‐ and TRPC1‐dependent phagocytosis. Viability of 4T1 cells co‐cultured with RAW264.7 macrophages 24 h post‐PEMF, assessed by (A) CyQuant (DNA content) and (B) MTT (metabolic activity) (*n* = 4 with 4 technical replicates each). (C) Representative images of colony‐forming assay on day 7 and (D) quantification of (i) total area and (ii) number of colonies (*n* = 3 with 3 technical replicates each). (E) Flow cytometry analysis of co‐cultures represented as (E) dot plots and (F) cell proportions shown as (i) stacked or (ii) separated bars. (*n* = 3 with 2 technical replicates each). (G) Representative scatter dot plots and the corresponding quantification (H) of phagocytosis of 4T1 by RAW264.7 macrophages assayed by flow cytometry, showing (i) no CSFE control, (ii) DMXAA (20 μg/mL), (iii–iv) 0 versus 3 mT PEMF; (iii); (v–vi) ± PEMF with STING inhibitor (2 μM C176); (vii–viii) ± PEMF with streptomycin. (*n* = 4 with 3 technical replicates each). (I) 4T1 viability in co‐cultures with phagocytosis inhibitor (1 μM Cytochalasin B), TRPC1 inhibitors (5 μM SKF‐96365 or streptomycin), STING inhibitor (2 μM C176), IRF3 inhibitor (0.1 μM BX795) or cGAS‐KO macrophages. (*n* = 3 with 8 technical replicates each). (J) (i) Representative western blots and (ii) quantification of CD47 expression on 4T1 cells treated with PEMF‐conditioned media from RAW264.7 macrophages. Data represent the mean ± the standard error of the mean (SEM). Statistical analysis was performed using Student's unpaired *t*‐test (D, J) or two‐way (A, B, F, H, I) ANOVA, followed by Šidák's multiple comparison post hoc test with *, *p* ≤ 0.05; **, *p* ≤ 0.01 and ***, *p* ≤ 0.001.

To simulate the TME in vitro, 4T1 cells and RAW264.7 macrophages were either cultured in isolation or in co‐culture at defined ratios. RAW264.7 macrophages were seeded at densities of 125, 250, and 500 cells per well relative to a fixed density of 1000 4T1 cells per well. PEMF exposure significantly reduced cell viability in co‐culture, as ascertained by CyQuant DNA staining 24 h post‐treatment, while monocultures of RAW264.7 or 4T1 cells were unaffected by PEMF exposure, indicating a context‐dependent effect (Figure 5A). Similarly, PEMF exposure revealed a significant decrease in metabolic activity (MTT assay) in co‐cultures when RAW264.7 cells were seeded at 250 and 500 cells per well (Figure 5B), indicating that PEMF exposure impairs cancer growth in a macrophage‐dependent manner. To determine whether reduced viability stemmed from macrophage or cancer cell loss, clonogenic and flow cytometry (FACS) analyses were performed. In a 2:1 (4T1:RAW264.7) co‐culture, PEMF exposure significantly suppressed colony formation over a 7‐day period (Figure 5C,D). The clonogenic assay measures the capacity of individual adherent cells to proliferate and form clonal colonies. Colony formation would thus be predominantly a feature of the adherent 4T1 epithelial cells rather than of the macrophage population, despite a low risk of macrophage infiltration into the 2D colonies. To ascertain the cell type experiencing the loss of viability, cells were analyzed by FACS using a pan‐leukocyte marker (CD45) to distinguish CD45^+^ macrophages from CD45^−^ 4T1 cancer cells. PEMF exposure preferentially reduced the CD45^−^ 4T1 population relative to their unexposed controls (Figure 5E,F), confirming that the observed loss in viability was specific to the cancer cell population.

Phagocytic capacity is a key component of the anti‐tumor activity of M1‐polarized macrophages and is regulated by activation of the NF‐κB pathway [48]. To evaluate PEMF‐mediated enhancement of phagocytic function, a co‐culture system with CFSE (carboxyfluorescein succinimidyl ester)‐labeled 4T1 cancer cells and RAW264.7 macrophages was established. Phagocytosis was quantified by flow cytometric analysis of CFSE^+^CD45^+^ macrophages, with the phagocytosis index defined as the percentage of CD45^+^ macrophages containing internalized CFSE signal. Using 20 μg/mL DMXAA as a positive control, > 15% of CD45‐positive macrophages exhibited internalized CFSE‐labeled cancer cellular debris, significantly exceeding the baseline levels established by untreated 0 mT controls (Figure 5Gi–iii). Notably, 3 mT PEMF exposure increased the phagocytosis index by 60% (Figure 5Giv), an effect that was completely abrogated by pharmacological inhibition of either STING (2 μM C176) or TRPC1 (streptomycin) (Figure 5G,H). To further validate phagocytosis as the mechanism underlying cancer cell clearance, two control experiments were performed. First, to confirm that CFSE uptake required direct cell contact rather than resulting from uptake of extracellular vesicles or protein fragments, macrophages and cancer cells were separated by a semipermeable (0.4 μm) transwell membrane. In this setup, CFSE uptake was abolished (Supporting Information S1: Figure S5B), indicating that direct contact is necessary. Second, the co‐cultured cells were treated with Cytochalasin B (CyB) to disrupt actin‐dependent processes and block phagocytic function [49]. Pre‐treatment with 1 μM CyB for 1 h prior to PEMF exposure significantly attenuated PEMF‐mediated reduction in cancer cell viability (Figure 5I). Consistent with our phagocytosis data, both TRPC1 inhibition (5 μM SKF‐96365, Streptomycin) and STING blockade (2 μM C176) for 1 h prior to PEMF exposure reduced cancer cell killing in co‐culture (Figure 5I). Additionally, 4T1 cells co‐cultured with cGAS KO RAW264.7 macrophages remained fully responsive to PEMF exposure, exhibiting a significant reduction in viability, comparable to that of PEMF‐exposed wild‐type cells (Figure 5I). Furthermore, inhibition of IRF3 with BX‐795 did not impair PEMF‐induced effects (Figure 5I), suggesting that the pro‐phagocytic phenotype is driven by the NF‐κB branch of the STING axis. We next investigated whether conditioned media from PEMF‐exposed macrophages could modulate the expression of CD47 on 4T1 cells, which are commonly overexpressed in cancers to evade phagocytosis [50]. However, no significant changes in CD47 expression were noted (Figure 5J). Finally, all drug concentrations were carefully titrated to concentrations that did not affect baseline cell viability (Supporting Information S1: Figure S6A). Taken together, these results establish that PEMF exposure enhances macrophage‐mediated phagocytosis of breast cancer cells through a cGAS‐independent TRPC1‐STING‐NF‐κB‐dependent signaling pathway.

### 2.6 PEMF Exposure Enhances M1 Macrophage Polarization and Promotes Selective Recruitment of M1 Macrophages in Tumor Spheroids

**FIGURE 6:**
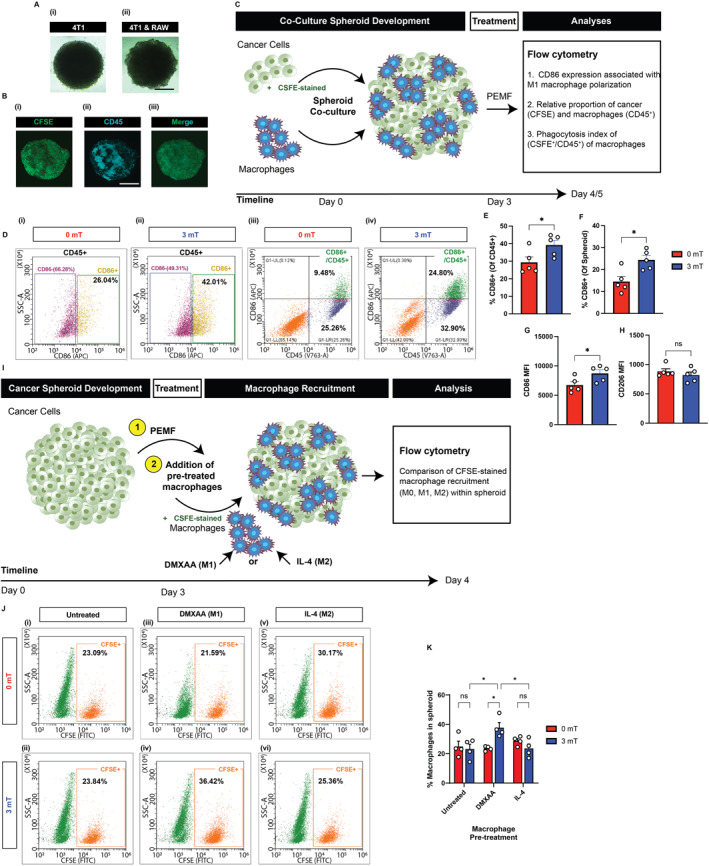
PEMF exposure enhances M1 macrophage polarization and promotes selective recruitment of M1 macrophages in tumor spheroids. (A) Brightfield images of 4T1 monoculture and 4T1‐RAW264.7 co‐culture spheroids. (B) Representative confocal microscopy images of 4T1‐RAW264.7 co‐culture spheroids: (i) 4T1 cells stained with CSFE (green), (ii) macrophages stained with CD45 (blue), and (iii) merged channels. Scale bar = 100 μm. (C) Schematic of the co‐culture spheroid development and experimental timeline for panels A–B and D–G. (D) Flow cytometry analysis of macrophages (CD45^+^) from co‐culture spheroids. Dot plots show: (i)–(ii) Gating for CD86^+^ macrophages within the CD45^+^ population, and (iii)–(iv) CD86^+^ macrophages within the total spheroid. (E, F) Quantified percentages of CD86^+^ macrophages within the CD45^+^ populations (E) and total spheroid (F), corresponding to plots in D. (G) Quantification of the median fluorescence intensity (MFI) of CD86 within the CD45^+^ macrophage population. (H) Quantification of the median fluorescence intensity (MFI) of CD206 within the CD45^+^ macrophage population. (I) Schematic of the pre‐polarized macrophage recruitment experimental timeline for panels J–K. (J) Flow cytometry analysis of CFSE‐labeled, pre‐polarized macrophage recruitment into 4T1 spheroids. Dot plots show incorporation of: (i)–(ii) Untreated (M0), (iii)–(iv) DMXAA‐treated (M1) and (v)–(vi) IL4‐treated (M2) macrophages. (K) Quantified percentage of recruited (CSFE^+^) macrophages within spheroids for each polarization condition, corresponding to plots in (J). Data are generated from *n* = 5 (D–H) or 4 (J–K) independent experiments with 3 technical replicates each (individual spheroids). Dots represent individual biological replicates. Values represent mean ± SEM. Statistical analysis was determined using Student's unpaired *t*‐test (E–H) or two‐way (K) ANOVA, followed by Šidák's multiple comparison post hoc test with *, *p* ≤ 0.05.

To investigate the effects of PEMF exposure in a more physiologically relevant context, we established a three‐dimensional (3D) co‐culture model to more closely replicate the TME. The hanging‐drop method [51] uses the force of gravity to promote cell aggregation in suspended droplets. This method was used to generate 4T1 monoculture spheroids and 4T1‐RAW264.7 co‐culture spheroids, as shown in the representative brightfield images (Figure 6Ai–ii). Successful co‐culture was confirmed by confocal microscopy, which showed CFSE‐labeled 4T1 cells (green) and CD45^+^ macrophages (blue) evenly dispersed throughout the entire spheroid structure (Figure 6Bi–iii). The impact of PEMF exposure on macrophage polarization was first assessed within this 3D TME (Figure 6C). Mirroring our 2D observations (Figures 1, 4), exposure of co‐culture spheroids to 3 mT PEMFs significantly increased the percentage of M1‐polarized (CD86^+^) macrophages, both within the CD45^+^ population and in the tumor spheroid (Figure 6D–F). Additionally, an increase in the bulk mean fluorescence intensity (MFI) of CD86^+^ macrophages was observed (Figure 6G), indicating a systemic shift toward the M1 phenotype. Conversely, the MFI for the M2 marker CD206 remained unchanged (Figure 6H). Next, the potential for PEMF pre‐treatment of cancer spheroids to influence the recruitment of differentially polarized macrophages was investigated (Figure 6I). 4T1 spheroids were exposed to PEMFs and subsequently incubated with CFSE‐labeled macrophages in M1‐like (DMXAA), M2‐like (IL‐4), or naive (M0) statuses. Flow cytometry analysis revealed that PEMF exposure did not alter the recruitment of naïve (M0) or IL‐4‐polarized (M2) macrophages (Figure 6Ji,ii,v,vi,K) after 24 h. By contrast, PEMF pre‐treatment selectively enhanced the incorporation of DMXAA‐polarized M1 macrophages into the tumor spheroids (Figure 6Jiii–iv,K). This finding aligns with our 2D chemotaxis data (Figure 4H–I) and further supports the notion that PEMF‐exposed tumors selectively recruit anti‐tumor macrophage subsets.

### 2.7 PEMF Exposure Increases Cancer Suppression and Promotes TRPC1‐STING‐ Dependent Phagocytosis in Tumor Spheroids

**FIGURE 7:**
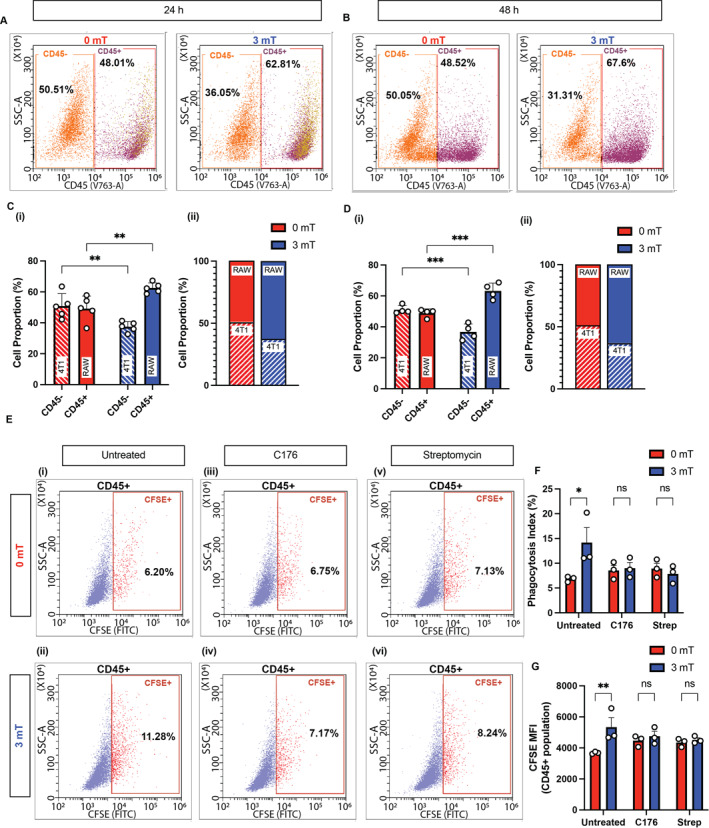
PEMF exposure drives tumor cell suppression and enhances TRPC1‐STING‐dependent phagocytosis in spheroids. (A, B) Representative flow cytometry dot plots analyzing cell populations within 4T1‐RAW264.7 co‐culture spheroids at 24 and 48 h post PEMF exposure, respectively. (C, D) Quantified cellular composition of spheroids corresponding to the plots in A and B, respectively. Data are presented as (i) separated or (ii) stacked bars showing the proportions of macrophages (CD45^+^) and cancer cells (CD45^−^). Data are from *n* = 4‐5 independent experiments, as indicated by dots in the bar charts, with 3 technical replicates each (individual spheroids). (E) Flow cytometry analysis of macrophage phagocytic activity. Representative scatter plots show the percentage of phagocytosing macrophages (CD45^+^CFSE^+^) under the following conditions: (i)–(ii) ± PEMF exposure, (iii)–(iv) ± PEMF exposure with STING inhibitor C176 (2 μM), (v)–(vi) ± PEMF exposure in the presence of TRPC1 inhibitor streptomycin (*n* = 3). (F) Quantification of the phagocytosis percentage, corresponding to the conditions in panel E. (G) Median fluorescence intensity (MFI) of CFSE within the CD45^+^ macrophage population, indicating phagocytic load. Dots represent individual biological replicates. Values represent mean ± SEM. Statistical analysis was performed using two‐way ANOVA, followed by Šidák's multiple comparison post hoc test with *, *p* ≤ 0.05; **, *p* ≤ 0.01, *** and *p* ≤ 0.001.

Having established that PEMF exposure promotes macrophages: (1) M1‐polarization; (2) pro‐phagocytic status; and (3) recruitment into tumors, we next investigated whether PEMF exposure translated into functional anti‐tumor activity. To this end, the direct phagocytic capacity of macrophages within PEMF‐exposed co‐culture spheroids was assessed. Flow cytometry analyses revealed a shift in the proportion of CD45^−^ cancer cells from 50% to 35% at 24 and 48 h post‐PEMF exposure (Figure 7A–D), indicating selective cancer loss. This was accompanied by a significant increase in phagocytic activity, determined by the percentage of CD45^+^CFSE^+^ macrophages (Figure 7Ei,ii,F) and the median fluorescence intensity of phagocytosed CFSE (Figure 7G, untreated). PEMF‐enhanced phagocytosis was attenuated by inhibition of either STING (C176) or TRPC1 (streptomycin) (Figure 7E–G), again confirming the contribution of this signaling axis in the observed tumor suppression.

### 2.8 PEMF Exposure Stalls Tumor Growth and Promotes Tumor Clearance In Vivo

**FIGURE 8:**
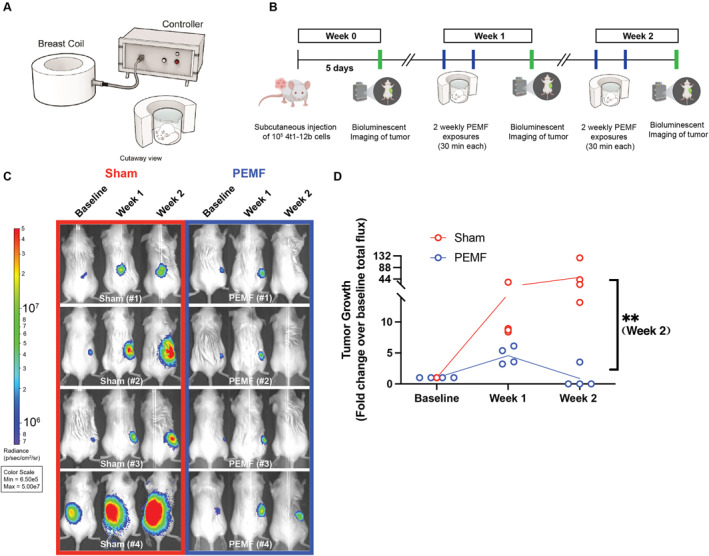
PEMF exposure suppresses tumor growth and promotes tumor clearance in vivo. (A) Schematic representation of the PEMF device used for animal exposure; the cutaway view illustrates the placement of the animal within the coil. Image adapted under terms of the CC‐BY license [21]. Copyright 2021, The Authors, published by Frontiers Media SA. (B) Experimental timeline detailing subcutaneous 4T1‐12b tumor induction, treatment regime (3 mT, 30 min, twice weekly), and periodic bioluminescent monitoring intervals. (C) Representative longitudinal IVIS bioluminescent imaging of BALB/c at baseline (day 5 post tumor injection), week 1 (day 12) and week 2 (day 19). (D) Quantitative analysis of tumor progression expressed as total flux (photons/sec) normalized to individual baseline values. Each dot represents single animals (*n* = 4 per group). Data are presented as mean ± SEM. Statistical analysis was performed using two‐way ANOVA, followed by Šidák's multiple comparison post hoc test with **, *p* ≤ 0.01.

Finally, the anti‐tumor activity of PEMF exposure was assessed in an immunocompetent murine model bearing subcutaneous 4T1‐12b breast tumors. Tumor establishment was confirmed 5 days post‐inoculation via IVIS bioluminescent imaging prior to the commencement of treatment. Mice were subsequently randomized to receive sham or 3 mT PEMFs twice weekly for 30 min and monitored by weekly IVIS imaging (Figure 8A–B). After 1 week (two exposures), tumors in both the sham and PEMF groups exhibited growth relative to baseline, although the tumors in the PEMF group showed an attenuated growth rate (4.5‐fold increase over baseline) compared with the sham control (14.5‐fold increase) (Figure 8C–D). Importantly, after two weeks of treatment (four total exposures), 3 of the 4 mice in the PEMF treatment group (75%) showed no detectable tumor bioluminescence, whereas the control group tumors were still growing (a 50‐fold increase over baseline). Subsequent macroscopic examination of the carcasses from the three corresponding PEMF‐treated mice showed no residual tumor remnants or fibrotic scarring. The single remaining tumor in the PEMF treatment group had also shrunk, decreasing from a 6‐fold baseline volume at week one to 3.5‐fold at week two. At the conclusion of the study, all the surviving tumors were harvested and subjected to TME profiling by flow cytometry (Supporting Information S1: Figure S7A–E). While 3 of 4 of the PEMF‐treated cohort achieved complete pathological clearance, the single remaining tumor in this group provided an opportunity for a mechanistic snapshot of the active immune remodeling driving the PEMF response. Compared with the four control tumors, this regressing PEMF‐treated tumor showed a profound enrichment of critical immune infiltrates, including M1‐polarized macrophages, CD8^+^ T cells, CD4^+^ T cells, natural killer T (NKT) cells, and natural killer (NK) cells. Furthermore, both NK cells and inflammatory monocytes within the PEMF‐treated TME exhibited elevated levels of activation markers (Supporting Information S1: Figure S7B,E). These data suggest that the observed tumor regression reflects the final stages of the macrophage‐polarizing and immune‐activating events that culminate in the eradication of 3 of the 4 PEMF‐treated tumors.

## 3 Discussion

The tumor‐microenvironment (TME) is a complex ecosystem comprised of diverse cell types and their cross‐modulatory interactions. In a pathological context, tumor‐resident cancer cells are known to co‐opt immune cells, thereby enabling tumor progression [43]. The use of immune checkpoint inhibitors (ICIs) is one therapeutic strategy aimed at counteracting this form of immunosuppression by blocking key inhibitory receptors (e.g., PD‐1, CTLA‐4) on the surface of T cells or their complementary ligands (e.g., PD‐L1) on the surface of cancer cells. ICIs thus disrupt the cancer‐mediated suppression of T cell function, restoring CD8^+^ cytotoxic T cell function and the ability of the immune system to seek out and eliminate tumors [43]. The clinical successes and FDA approval of ICI‐based strategies are clear evidence of the translational value of therapies aimed at reprograming the TME.

Chemotherapy remains the cornerstone of breast cancer treatment [21]. Chemotherapy can directly kill cancer cells as well as modulate the TME toward tumor repression [52]. On the other hand, chemotherapy comes with significant risks of developing chemoresistance and associated side effects [53]. Immune cells can be metabolically hijacked within the TME to promote chemoresistance in cancer cells. For instance, TAM‐derived IL‐10 has been shown to drive breast cancer resistance to doxorubicin and paclitaxel [54] and to accelerate tumor progression [55]. On the other hand, strategies designed to overcome chemoresistance, such as combinatorial therapy [56] or dose escalation [57], often exacerbate chemotherapy‐related toxicity. Given the multidimensional manner in which the TME reacts to sustain tumor progression, there is a clear need for the development of alternative therapies that comprehensively modulate the TME without compounding systemic toxicity burden.

Macrophage polarity is subject to modulation by biophysical stimuli such as light, electricity, or magnetism. These forms of biophysical stimuli, by mere virtue of the fact that they elicit cellular responses, will ultimately impinge upon mitochondrial activity [14], which in turn, will modulate macrophage polarization [58]. Cryptochromes are a family of magneto‐sensitive photoreceptors that, when examined, have been shown to interact with a TRPC1‐mitochondrial signaling axis [30] to regulate cell cycle progression [15]. Accordingly, red and near‐infrared (NIR) light have been shown to promote phagocytosis and induce anti‐inflammatory cytokine secretion in a dose‐dependent manner downstream of mitochondrial activity [59]. Magnetic fields are also capable of polarizing macrophages into either the M1 or M2 statuses, depending on specific parameters of the applied fields [60]. Finally, square and sinusoidal electrical waveforms have been shown to enhance M1 and M2 polarizations, respectively, via an electrogenic effect on the cell membrane potential and intracellular calcium levels that, in turn, modulate the expression of polarization‐associated receptors such as TLR4 and IL‐4R*α* [61]. On the one hand, photonic and electrical stimulations face practical challenges. NIR light exhibits poor tissue penetration [62] and is readily absorbed by fat [63], making it particularly unsuitable for treating tumors within the breast. Electric stimulation, by virtue of the fact that its delivery to an implicated tissue region requires direct contact with electrodes, has a greater propensity to cause general tissue damage [64] while also being invasive [65]. On the other hand, organic tissues such as bone, fat, and muscle pose no barrier to the penetration of extremely low‐frequency PEMFs, enabling uniform targeting into deep anatomical sites [66, 67]. Thus, PEMFs can be delivered to a tumor embedded deep within a breast without requiring direct contact with the overlaying tissue, minimizing patient discomfort and procedural risks while facilitating repeated exposures. Well‐designed PEMF systems can generate uniform magnetic fields within a targeted tissue region, such as the breast, which is crucial for achieving predictable biological effects and reaching underdosed areas with limited perfusion, thereby mitigating potential compromises in therapeutic efficacy. These properties make PEMF‐based therapies a promising non‐invasive modality for modulating the TME in deep‐seated tumors.

A growing body of evidence now clearly indicates that magnetic fields can modulate cell responses [66]. Immunologically, electromagnetic exposure has been shown to exert immunomodulatory effects across immune cell populations, including B cells, T cells, macrophages, neutrophils, and NK cells [68, 69]. Macrophage responses are sensitive to features of the magnetic signal [60] that differentially modulate mitochondrial function through an adaptive process known as magnetic mitohormesis [14, 15]. A mitochondrial hormetic response would explain the context dependence of magnetic therapy: why brief exposure to low‐intensity magnetic fields promotes beneficial mitochondrial adaptive responses, whereas higher intensity exposures for longer durations may prove detrimental to mitochondrial function [70]. Previous studies have reported that PEMF exposure in the microTesla (μT) [60] to low‐milliTesla (1.5 mT) [71] range promotes anti‐inflammatory responses in macrophages. The magnetic mitohormetic nature of electromagnetic exposure invokes the requirement of optimizing magnetic field parameters such as amplitude, frequency, and duration of exposure for the most effective channeling of cellular responses [14, 15]. Coupled with their non‐invasive and localizable delivery, the tunability of magnetic stimulation makes them a promising therapeutic modality for immune modulation. We previously developed and validated a 3 mT PEMF paradigm that was effective in slowing breast cancer growth, both as a standalone [20] treatment and in combination with chemotherapy [21, 22], particularly with respect to TRPC1 expression [21, 22]. Specifically, 10 min exposure to 3 mT PEMFs was later shown to enhance doxorubicin uptake via a TRPC1‐mediated mechanism [22]. Here, we build upon these previous findings by showing that 3 mT PEMF exposure is also capable of modulating TAMs toward an anti‐tumor status dependent on TRPC1 expression.

The TRP channel superfamily mediates critical roles in cellular development, sensory and signal transduction and is organized into several subfamilies that respond to various forms of biophysical stimuli [72]. TRPC1 was the first mammalian TRP channel characterized and has the highest degree of homology to the original *Drosophila* TRP channel. TRPC1 has emerged as a particularly promising target in cancer, where its overexpression correlates with tumor progression by promoting proliferation, epithelial‐mesenchymal transition, and invasiveness [16, 21]. TRPC1 serves as a regulator of the other TRPC family members via a process of selective heteromultimerization [73, 74], theoretically uniting their distinct biophysical activation modes into a single‐channel complex, yet complicating their pharmacological mechanistic dissection. Upregulation of TRPC1 has been reported in breast, colorectal, lung and pancreatic cancers, often correlating with more aggressive forms of the cancer associated with poorer survival outcomes [16]. These features position TRPC1 as a promising prognostic marker and therapeutic target for precision cancer interventions.

TRPC1 has now been convincingly shown to be a key member of a magnetoreception complex that plays critical roles during mitochondrial‐modulated development or cancer progression. TRPC1 ablation abolished PEMF‐induced myogenic progression [15, 30], whereas TRPC1 overexpression enhanced the vulnerability of breast cancer cells to combined PEMF and doxorubicin treatments [21, 22]. As macrophage polarization has been shown to be regulated by TRPC1‐mediated Ca^2+^‐signaling [13], PEMF‐mediated responses in macrophages were examined in the context of TRPC1 function and expression. TRPC1 was found necessary for PEMF‐mediated responses in RAW264.7 macrophages and 4T1 breast cancer cells as genetic silencing or pharmacological blockade of TRPC1‐mediated calcium entry attenuated PEMF‐mediated phosphorylation of NF‐κB (Figure 3A,B,I, Supporting Information S1: Figure S4). Accordingly, PEMF‐mediated calcium entry was blocked by a broad spectrum of TRPC1 channel antagonists, including Pico145 (also known as C31 or HC‐608) of the highest available specificity [75], but not by antagonists to other candidate TRP channel species, including TRPA1 and TRPV4 (Figure 3I, Supporting Information S1: Figure S4). These results align with in vitro and in vivo models, in which genetic and pharmacological disruption of TRPC1 impaired the expression of key M1‐associated inflammatory mediators [13].

Here, we provide evidence that TRPC1 and STING activation are mechanistically coupled. The activation of the STING pathway is recognized for its anti‐tumoral effects in both cancer cells and macrophages [76]. Cancer cells often produce damaged DNA, which activates the cGAS‐STING cascade, driving cancer immunogenicity and orchestrating the coordinated reprogramming of macrophages alongside the recruitment of effector populations, including T and NK cells [76]. Apart from balancing efficacy and systemic toxicity, clinical translation of STING agonism may be hindered by the fact that physiological outcomes depend on the kinetics and duration of stimulation. Transient activation of STING promotes canonical NF‐κB‐mediated immune activation and tumor clearance [77], whereas chronic activation paradoxically drives pro‐tumorigenic pathways by dampening immune responses and promoting non‐canonical NF‐κB signaling [78]. This temporally defined response disparity would require the precise control over stimulation duration, sufficient to elicit tumor immunity, but without tipping the balance into pro‐tumorigenic chronic STING activation. It is thus of clinical relevance that PEMF‐based modalities enable exact spatiotemporal control of STING activation, potentially capable of harnessing its anti‐tumor effects while avoiding chronic overactivation. PEMF‐based therapies thus offer a level of control not readily available with drug‐based approaches that are limited by pharmacokinetic and pharmacodynamic considerations imposed by dead spaces within tissues and limited penetration due to abnormal vascularization of tumors [79].

In this study, we demonstrated that a single 10‐min exposure to 3 mT amplitude PEMFs, applied in the extremely low frequency range (15 Hz), was sufficient to induce M1‐like polarization of both RAW264.7 macrophages and bone marrow‐derived macrophages (BMDMs). These findings are consistent with the hypothesis that PEMF exposure induces STING‐dependent M1‐like macrophage polarization (Figure 1), whereby STING triggers NF‐κB‐mediated upregulation of iNOS and CD86 while suppressing Arginase expression [26, 80, 81]. The reduction in M2 marker expression (Figure 1) coupled with an increase in bulk expression of M1 markers (Figures 1, 6) and an increase in proportion of M1 macrophages (percentage CD86^+^, Figure 6E–F) all support that PEMF exposure increases M1 status through the repolarization of M2 macrophages. M1‐like reprogramming, in turn, was correlated with a significant increase in phagocytic clearance of breast cancer cells by said macrophages. Furthermore, these findings align with existing reports showing that PEMF exposures in the 15–30 Hz and 3–4 mT ranges trigger NF‐κB activation in immune cells, including T cells [82]. Mechanistically, we demonstrated that PEMF‐induced NF‐κB activation requires STING independent of cGAS. Genetic ablation of cGAS did not impair PEMF‐driven NF‐κB activation in isolated macrophages or co‐culture viability assays. Moreover, pharmacological antagonism of STING (or TBK1) as well as genetic ablation of TRPC1 (or STING) abrogated PEMF‐mediated NF‐κB phosphorylation (Figure 2, 3) and PEMF induced oligomerization of STING (seen as dimers in non‐reducing SDS‐PAGE, Figure 2C), a critical step in activation of the STING pathway [83].

Secretome harvested from PEMF‐treated 4T1 cancer cells enhanced NF‐κB signaling, iNOS and CD86 expression in RAW264.7 macrophages (Figure 4E,F) as well as promoted the selective chemotaxis of M1‐polarized macrophages to cancer cells (Figure 4H,I). On the other hand, PEMF conditioned media from cancer cells or DMXAA treatment of macrophages alone did not promote macrophage recruitment. One explanation for this observation is that simultaneous expression of chemokine receptors in macrophages (induced by M1 polarization‐DMXAA treatment) [46] and elevated chemokine release by cancer cells (induced by PEMF) work in unison to enhance macrophage recruitment. Non‐invasive and highly penetrative PEMF exposure may thus subvert the immunosuppressive drive of the TME through a combination of comprehensive macrophage polarization and cancer cell‐mediated crosstalk.

**FIGURE 9:**
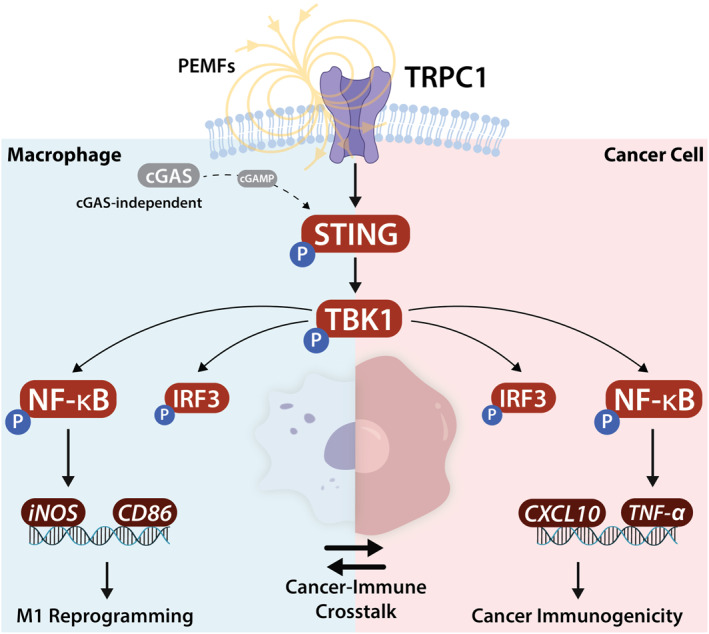
Schematic illustration of PEMF‐induced, cGAS‐independent, TRPC1‐STING‐NF‐κB activation resulting in macrophage reprogramming and enhanced cancer immunogenicity.

PEMF exposure may synergize with DMXAA (STING agonist) in macrophages to amplify immune activation for enhanced cancer clearance. Although conditioned media from PEMF‐exposed RAW264.7 macrophages had no effect on cell growth of 4T1 breast cancer cells (Supporting Information S1: Figure S6B), direct co‐culture experiments revealed a PEMF‐dependent reduction in cancer viability. This reduction was accompanied by the accumulation of phagocytosed 4T1‐derived material within macrophages, consistent with stimulated phagocytosis (Figures 5, 7). Specificity for cancer cells was demonstrated by showing that co‐culturing of macrophages with non‐cancerous C2C12 myoblasts produced no signs of cell deterioration (Supporting Information S1: Figure S6C,D). The developmental attributes of carefully monitored PEMF exposure on non‐malignant cell types have been reported in the literature [66]. It is important to clarify at this point that the selectivity of the reported anti‐cancer response appears to be predominantly attributable to the characteristics of macrophage cellular machinery rather than to a generalized cytotoxic effect of magnetic fields per se. While the phagocytosis of synthetic materials by macrophages in response to PEMF exposure has been reported [84], we uniquely demonstrated the specific targeting of cancer cells for phagocytosis by PEMF‐polarized macrophages (Figure 9). Our results further align with previous studies showing that STING agonism promotes cancer phagocytosis [85], whereas STING inhibition abrogates phagocytosis (Figure 5G,I). Moreover, because neither the conditioned media from PEMF‐exposed macrophages (Supporting Information S1: Figure S6B) nor direct exposure of cancer cells in isolation (Figure 5A,B) influenced cancer viability, the observed PEMF‐induced effect likely required direct contact between macrophages and cancer cells. Accordingly, inhibiting phagocytosis using Cytochalasin B [49] completely abolished PEMF‐induced cytotoxicity in co‐culture. Taken together, the data suggest that macrophage phagocytosis is the principal mode of anti‐tumor immunity induced by PEMF exposure in cancer‐macrophage co‐cultures.

NF‐κB activation in the context of cancer is a proverbial double‐edged sword. While it is crucial in orchestrating cytotoxic immune responses, NF‐κB activation has also been shown to enhance anti‐apoptotic factors and promote survival [86]. Numerous factors affect how NF‐κB activation influences cancer outcomes. For example, the concomitant activation of the STAT3 transcription factor synergizes with NF‐κB to drive tumor progression [86]. On the other hand, emerging evidence suggests that STING‐dependent NF‐κB activation promotes tumor clearance [26], aligning with our demonstrated PEMF‐induced STING‐dependent NF‐κB activation producing tumoricidal responses. Therefore, the manner of NF‐κB activation determines its functional outcome. This result thus distinguishes PEMF paradigms from potentially pro‐tumorigenic mechanisms acting independently of STING‐dependent NF‐κB activation.

Amongst the many strategies aimed at targeting macrophage polarization, STING agonism has emerged as a preferred candidate [12]. This potential translational bias is due to the documented synergy between STING activation and existing treatments, such as chemotherapy (cisplatin, doxorubicin, and carboplatin) and other targeted therapies (PD‐1/PDL‐1 blockade and PARP inhibitors) [29]. On the other hand, the clinical translation of STING stimulatory therapies has been hampered by concerns of heightened toxicity [12]. Intelligently designed PEMF modalities may thus represent minimally invasive adjunct therapies that better harness the therapeutic benefits of STING while avoiding the toxicity burden often associated with pharmacological STING agonists.

Cancer‐macrophage spheroids emulated key aspects of the PEMF‐induced interactions evident between macrophages and cancer cells observed in 2D. Specifically, spheroids recapitulated the recruitment of M1‐activated macrophages and the enhanced phagocytosis of cancer cells induced by PEMF exposure. Validation in the spatially structured environment provided by spheroids added substantial translational value to the study as they more closely approximate the physical barriers, nutrient gradients, and cell‐cell contacts of the native TME. The spheroid model enabled analysis and observation that would have been difficult in animal models.

Finally, the here‐presented PEMF paradigm was validated in immunocompetent mice bearing aggressive 4T1 tumors. Remarkable therapeutic efficacy was observed, with 3 out of the 4 PEMF‐treated mice achieving complete pathological resolution after just 2 weeks of treatment (4 exposures). In contrast, untreated mice exhibited exponential tumor growth under identical housing conditions. The same PEMF device was previously shown to attenuate tumor growth in an immunocompromised (NSG) patient‐derived xenograft (PDX) breast cancer mouse model; the study reported slower overall tumor resorption rates, though the magnitude of the effect was comparable to that of doxorubicin chemotherapy [21]. While PEMF treatment can stall tumor progression in an immunocompromised model via direct effects on cancer cells [21], the superior regression and clearance of tumors in the current immunocompetent model confirms the role of the immune system in mediating the curative potential of this therapy. Although only a single PEMF‐treated tumor remained for analysis, ex vivo profiling revealed a profound shift in the immune landscape that mirrored our in vitro findings. The clear expansion of M1 macrophages (but not M2), CD8^+^ T cells, CD4^+^ T cells, NK cells, NKT cells, B cells and activated inflammatory monocytes is in line with the observed tumor clearance, demonstrating the potential for PEMF treatment to alter the TME to facilitate immune‐mediated tumor clearance. Clinically, this coil technology has recently completed Phase 1 safety trials in cancer patients with no adverse effects reported (ClinicalTrials.gov ID: NCT06332508). Taken together, these findings highlight the potential of PEMF therapy as a non‐invasive adjuvant to existing immunotherapeutic strategies.

## 4 Limitations

While this study unraveled the PEMF‐TRPC1‐STING‐NF‐κB axis in macrophage‐cancer crosstalk, it focused solely on these two resident cell types in the TME. We did not examine the influence of PEMF exposure on other critical immune populations, such as T and natural killer (NK) cells, whose recruitment and activity are vital for a complete anti‐tumor immune response. Future work should characterize the broader immunomodulatory landscape of PEMF stimulation. While the presented in vivo model helped address this question initially, it also comes with caveats that require careful consideration. Given the small size of the murine model, isolating the tumor for PEMF exposure was not feasible, resulting in unavoidable exposure of collateral tissues and their respective responses to magnetic fields. Comparing tumor shrinkage in wild‐type mice versus macrophage‐depleted mice exposed to PEMFs, or not, would represent one approach to reveal the contribution of macrophages to the overall effect of systemic PEMF exposure. A critical caveat of this proposed experimental design is that the measured effect would then reflect the systemic impact of depleting all macrophages within the animal and not the role of resident TAMs per se. In principle, isolated breast tumor exposure has been achieved in Phase 1 human clinical trials using this magnetic platform (ClinicalTrials.gov ID: NCT06332508).

## 5 Conclusion

The findings presented in this study expand upon prior work as well as delve further into the potential implications of magnetic mitohormesis in modulating the interactions between macrophages and breast cancer cells. Brief PEMF exposure (10 min, 3 mT) was shown to polarize macrophages toward an anti‐tumoral M1‐like phenotype. A cGAS‐independent STING activation pathway was identified that was responsive to a PEMF signaling axis requiring the participation of TRPC1. This PEMF‐TRPC1 signaling pathway stimulated the phagocytic capacity of macrophages in co‐culture, spheroids and animal models, revealing a dynamic interplay between cancer and immune cells that was mechanistically geared toward ultimate tumor repression, independent of whether either cell type was exposed separately or in combination. On the technical level, the spatial precision achievable by targeting magnetic fields to specific anatomical area positions PEMFs as a promising immunotherapeutic strategy. The ability to non‐invasively direct pro‐phagocytic, M1‐polarizing signals to a tumor could enable localized immune activation. Future clinical validation is warranted for the potential of PEMF‐based therapies to effectively remodel the tumor microenvironment in humans and to serve as an adjuvant therapy for cancer immunotherapy.

## 6 Experimental Methods

### 6.1 Cell Culture and Pharmacological Reagents

RAW264.7 macrophages and C2C12 murine myoblast cells were purchased from ATCC and maintained in Dulbecco's Modified Eagle Medium (DMEM) (Thermo Fisher Scientific, Waltham, MA, USA) and 10% fetal bovine serum (FBS) (Biowest, Nuaillé, France). 4T1 and 4T1‐12b breast cancer cells were purchased from ATCC and adapted to grow in DMEM supplemented with 10% FBS via a stepwise transition protocol beginning with an initial culture in a 1:1 (DMEM/RPMI) for several weeks, followed by a complete transition to DMEM. Throughout the adaptation period, the cells maintained normal proliferation and morphological characteristics compared with those cultured in native RPMI medium. cGAS Knock‐out RAW264.7 cells (described below) were maintained in DMEM with 10% FBS. All cell lines were passaged every 3 days or once 80% confluence was reached. Bone marrow‐derived macrophages (BMDMs) were isolated from C57/BL6 mice as per a previously established protocol [44]. All cell lines were maintained at 37°C and 5% CO_2_.

For polarization experiments, RAW macrophages were pre‐treated with IL‐4 for 24 h prior to seeding at 50,000 cells/mL. RAW macrophages were seeded at a density of 100,000 cells/mL for the time‐course and inhibitor experiments, and 25,000 cells/mL for the siRNA knockdown experiments. 4T1 cells were seeded at a density of 100,000 cells/mL for the Western Blot and qPCR analysis. For the co‐culture assays, 4T1 (10,000 cells/mL) and RAW (5000 cells/mL) cells were thoroughly mixed in equal volumes prior to seeding for FACS analyses and MTT and CyQuant assessments. The cells were seeded and allowed to attach overnight in tissue culture dishes for all Western Blot, qPCR, and FACS protocols with 2 mL and 100 μL of media per well for 6‐well and 96‐well plates, respectively, for CyQuant and MTT assessments. Seeding densities were decided based on the number of days post‐seeding till harvest to avoid cell overgrowth.

IL‐4 (100 ng/mL) was purchased from Gibco and MCC950 (0.5 μM) was purchased from InvivoGen. AP‐18 (10 μM), RN‐9893 (1 μM), 5,6‐dimethylxanthenone‐4‐acetic acid (DMXAA) (20 μg/mL), C176 (2 μM), and Amlexanox (100 μM) were purchased from MedChemExpress. Cytochalasin B (1 μM), Streptomycin (100 mg/L), Gentamicin (50 mg/L), 1X Penicillin‐Streptomycin (Pen‐Strep), Neomycin (50 mg/L), 2‐Aminoethoxydiphenyl borate (2‐APB) (10 μM), N‐acetylcysteine (NAC) (5 mM) and SKF‐96365 (5–50 μM) were purchased from Sigma‐Aldrich.

### 6.2 Spheroids

Spheroids composed of 4T1 monoculture or in co‐culture with RAW264.7 were generated using a modified hanging drop method [87, 88]. Briefly, 4T1 and RAW264.7 cells were adjusted to 2 × 10^6^ cells/mL. For co‐culture spheroids, the two cell suspensions were mixed in a 1:1 volume ratio. 20 μL of the respective cell suspensions were seeded onto the inverted lid of a 2 mL petri dish (9 droplets per dish). The lid was carefully inverted and placed over the corresponding Petri dish containing 2 mL of PBS to maintain humidity. Spheroids were cultured on an orbital shaker (70 RPM) at 37°C with 5% CO_2_ throughout formation and subsequent experimentation. From day 0 to day 3, the culture media were partially exchanged daily by carefully replacing 10 μL of spent medium with fresh media using a 10 μL micropipette tip. All treatments (PEMF exposure/drug treatment) were administered directly within the hanging drop on day 3. Following treatment, spheroids were maintained on the orbital shaker until harvest. 4T1 only spheroids were generated for the macrophage incorporation assay. On day 2, pre‐seeded macrophages were treated with DMXAA or IL‐4 for 24 h. On day 3, 10 μL of media was replaced with 5 μL fresh media in the hanging droplets. Pre‐treated macrophages were harvested and stained with CFSE (described below) and adjusted to a concentration of 2 × 10^6^ cells/mL while the 4T1 cancer spheroids underwent PEMF exposure. Immediately after exposure, 5 μL of the RAW264.7 cells was added to the droplets, and the spheroids were placed in the shaker for 24 h prior to harvesting. For flow cytometry analysis, spheroids were washed twice in PBS to remove any free‐floating cells, visually inspected under the microscope, and then collected and dissociated with trypsin. The resulting single‐cell suspension was centrifuged at 2000 RPM for 5 min and prepared for FACS as described below.

### 6.3 CRISPR/Cas9 cGAS Knockout RAW264.7 Macrophages

cGAS‐deficient RAW264.7 cells were generated using the CRISPR‐Cas9 system as previously described [89]. Briefly, a cGAS U6‐gRNA vector encoding a guide RNA targeting exon 1 of the *cGAS* gene (GGCCCCCATTCTCGTACGGAGGG) was co‐transfected with the Cas9‐2A‐GFP plasmid (pSpCas9(BB)‐2A‐GFP, Addgene #48138) into RAW264.7 cells using Transfectin (Biorad). 24 h post‐transfection, GFP‐positive cells were isolated using a single‐cell sorter into 96‐well plates for clonal expansion. cGAS knockout was validated by Sanger sequencing and immunoblotting for cGAS protein expression.

### 6.4 Genetic Silencing of STING, STIM1 and TRPC1

Transient knockdown of STING, STIM1 or TRPC1 was achieved using two pre‐designed dicer‐substrate short interfering RNAs (dsiRNA) per target (IDT). dsiRNAs were designed against the coding sequence of mouse STING (NC_000084.7), STIM1 (NC_000073.7) or TRPC1 (NC_000075.7). RAW264.7 cells were transfected with of 1 nM (STING) or 10 nM (STIM1) dsiRNA using Lipofectamine 3000 reagent (Invitrogen) according to the manufacturer's protocol. For TRPC1 silencing, seeded RAW264.7 cells were serum‐starved for 24 h and switched to DMEM supplemented with 2.5% FBS 1 h prior to transfection with 1 nM dsiRNA using Lipofectamine 3000. Knockdown efficiency was validated 24 h (STING/STIM1) or 48 h (TRPC1) post‐transfection by western analysis, comparing protein levels to those in cells transfected with a negative control (NC) dsiRNA. All dsiRNA sequences are provided in Supporting Information S1: Table S1.

### 6.5 Western Analysis

Total protein extraction was carried out using RIPA lysis buffer containing 50 mM NaCl, 1 mM EDTA, 50 mM Tris‐HCl, 1% Triton X‐100, 0.05% SDS, 1 × protease inhibitor (Thermo Fisher Scientific), and 1x phosphatase inhibitor (Thermo Fisher Scientific). Lysates were collected using a cell scraper and incubated at 4°C for 30 min before being spun at 12,000 RPM for 15 min at 4°C. Total protein quantification was done using a Pierce BCA protein assay kit (Thermo Fisher Scientific). Protein samples with 1 × loading dye (Bio‐Rad) with β‐Mercaptoethanol (Sigma‐Aldrich) were boiled at 95°C, resolved on SDS‐PAGE, transferred to a PVDF membrane (Thermo Fisher Scientific) and blocked with 5% BSA (Nacalai Tesque) for 1 h. Samples for non‐reducing SDS‐PAGE were similarly treated with 1x loading dye but without β‐Mercaptoethanol and without boiling lysis. Blots were incubated with primary antibodies overnight at 4°C and with species‐specific secondary antibodies for 1 h at room temperature. Blots were imaged on Odyssey Fc (Li‐COR Biosciences) and analyzed using ImageStudioLite software (Li‐COR Biosciences). List of antibodies used is found in Supporting Information S1: Table S2.

### 6.6 Calcium Imaging

Calcium Imaging was performed using Calcium Green‐1 AM (Invitrogen) following the manufacturer's protocol. Briefly, 5000 cells per well were pre‐seeded into 96‐well plates. Cells were loaded with 5 μM calcium green dye in FluoroBrite DMEM (Gibco) for 1 h before washing thoroughly in FluoroBrite DMEM. TRP channel inhibitors were diluted in FluoroBrite DMEM, and cells were incubated with inhibitors for 15 min. Baseline was assessed for 2 min 30 s with 5 reads, 30 s apart at 506/531 nm on Cytation 5 microplate reader (BioTek) and plates were immediately exposed or not to PEMFs. Immediately after PEMF exposure, cells were returned to the plate reader for readings taken 30 s apart for 10 min. Each reading for each well was normalized by subtracting its corresponding baseline reading to obtain the true change in fluorescence. The average of 3 time‐point readings was used to plot each data point.

### 6.7 Real‐Time qPCR

Quantitative reverse‐transcription polymerase chain reaction (RT‐qPCR) was performed using a SYBR green‐based detection workflow. Briefly, total RNA was harvested from 4T1 cells using the RNeasy kit (Qiagen) and 0.5 μg of RNA was reverse transcribed to cDNA using the iScript cDNA Synthesis kit (Bio‐Rad). Quantification of gene transcript expression was performed using SSoAdvanced Universal SYBR Green (Bio‐Rad) on the CFX Touch Real‐Time PCR Detection System (Bio‐Rad). Relative transcript expression was determined using the 2^−ΔΔCt^ method, normalized to β‐2‐microglobulin (B2M) transcript levels. Primer sequences for qPCR are found in Supporting Information S1: Table S3.

### 6.8 Generation of PEMF Conditioned Media

Donor 4T1 cells were seeded at a density of 500,000 cells per T75 flask and allowed to adhere overnight. Pre‐plated cells were given 7 mL of fresh complete media 1 h prior to PEMF exposure. Conditioned media were collected 24 h after PEMF exposure and centrifuged at 1200 RPM for 5 min prior to treatment of recipient macrophages. Control (0 mT) conditioned media was collected in the same manner, except that the donor flask was placed in the PEMF device while in the off mode. Both control and PEMF‐treated cells were exposed to room temperature for 10 min while undergoing their respective treatments.

### 6.9 Invasion Assay

Invasion assay was performed using the CytoSelect 24‐well Cell Invasion Assay kit (Cell Biolabs Inc., San Diego, CA, USA) according to the manufacturer's protocol. Briefly, RAW264.7 macrophages pre‐treated with DMXAA and IL‐4 for 24 h were seeded (195,000 cells) in the cell culture insert after the rehydration of the basal membrane in FBS‐free media. The lower well of the invasion plate was filled with conditioned media from 4T1 after 24 h of conditioning. The setup was incubated for 48 h in a standard tissue culture incubator before the extraction and staining of the invaded cells from the basal membrane. Lysates from the extracted cells were analyzed at OD 560 using a Cytation 5 microplate reader (BioTek).

### 6.10 Determination of Cell Viability

Cell viability was assessed by measuring DNA content (CyQuant Cell Proliferation Assay kit, Thermo Fisher Scientific, Waltham, MA, USA) or by measuring metabolic activity (MTT Cell Proliferation Kit, Roche) according to the manufacturer's protocol. Briefly, RAW264.7 macrophages and 4T1 cancer cells were seeded at ratios of 1:8, 1:4 or 1:2 in a 96‐well format with 8 technical replicates per condition. The co‐culture was exposed to magnetic stimulation (3 mT) for 10 min and analyzed 24 h after.

For CyQuant, the plates were read at 480/520 nm on a Cytation 5 microplate reader (BioTek, Winooski, VT, USA). For MTT, plates were analyzed using the MTT labeling agent and solubilization solution at 600 nm on a Cytation 5 microplate reader (BioTek, Winooski, VT, USA).

### 6.11 Flow Cytometry

4T1 cells and RAW264.7 macrophages were seeded in a 2:1 ratio and the co‐culture was exposed to PEMFs 24 h later. CD45 was used to distinguish macrophages (CD45^+^) from tumor cells (CD45^−^). The specificity of CD45 staining in both RAW264.7 and 4T1 was assessed using single‐culture, single‐stained controls (Supporting Information S1: Figure S5A). Harvested cell pellets were blocked in Fc block (1:50) in FACS buffer (0.1% sodium azide in PBS). Cells were then incubated in BV711 Rat Anti‐Mouse CD45 antibody (1:200, BD Biosciences) for 1 h. Cells were washed three times in PBS and resuspended in FACS buffer. 10,000 events were recorded for each sample. Cell suspensions were filtered through a 40 μm filter to eliminate aggregates and ensure single‐cell suspension. Samples were analyzed on a CytoFLEX LX Flow Cytometer (Beckman Coulter), and the results were analyzed by CytExpert software (Beckman Coulter). CD45‐BV711 was excited at 405‐nm and detected in the V780 channel (780/60 filter), whereas CFSE was excited at 488‐nm and detected in the B525 channel (525/40 filter). Single‐stained controls confirmed negligible spillover and thus no further compensation was applied.

For the animal study, harvested tumors were digested in digestion buffer (1% FBS and 1 mg/mL Collagenase I in RPMI) for 2 h to achieve single‐cell dissociation. The single cell suspension was incubated in appropriate antibody cocktail (Supporting Information S1: Table S4) for 30 min in FACS buffer and washed twice prior to analysis on a CytoFLEX LX Flow Cytometer. Established gating strategies [90, 91, 92, 93] were used and are provided in Supporting Information S1: Table S5. Gates with fewer than 100 events were excluded from the analysis. Single‐stained beads were used to apply compensation (Supporting Information S1: Table S6–S7), and unstained samples were used for gating.

### 6.12 Phagocytosis Assay

A previously established method using CFSE staining of cancer cells was employed for the phagocytosis assay [94]. Briefly, 4T1 cancer cells were incubated in 1 μM CFSE (MedChemExpress) in PBS for 10 min prior to quenching in DMEM with 10% FBS and subsequently washed twice in DMEM with 10% FBS. The cells were incubated in DMEM with 10% FBS for 10 min prior to another two washes. The stained 4T1 cells were seeded in a 2:1 ratio with RAW264.7 macrophages. 48 h after PEMF exposure of the co‐culture, the culture was harvested and subjected to flow cytometry. CD45 was used to differentiate macrophages (CD45^+^) from tumor cells (CD45^−^). The specificity of CD45 and CFSE staining in both RAW264.7 and 4T1 was assessed using single‐culture, single‐stained controls. CFSE detected in CD45^+^ RAW264.7 macrophages was considered indicative of phagocytosis. CD45 was sufficient to distinguish macrophages from cancer cells without interfering with the CFSE signal. Single population controls of macrophages alone and CFSE‐stained cancer cells showed that CFSE did not interfere with CD45 staining (Supporting Information S1: Figure S5A). 10,000 events were recorded for each sample. Cell suspensions were filtered through a 40 μm filter to eliminate aggregates and ensure single‐cell suspension. A control experiment was conducted under identical conditions, except that a 0.4 μm pore‐size transwell insert (Costar) was used to separate the cell lines (Supporting Information S1: Figure S5B).

### 6.13 Colony‐Forming Assay

Clonogenic assay was performed using crystal violet staining. 100 4T1 cells and 50 RAW264.7 macrophages were seeded per well in a 6‐well plate format. Fresh complete DMEM was provided on days 1 and 4. Cells were exposed to 3 mT PEMFs for 10 min on day 1 only. On day 7, the cells were rinsed in PBS and stained with a crystal violet solution consisting of 0.5% crystal violet and 6% glutaraldehyde (Sigma‐Aldrich) in distilled water for 1 h. Stained colonies were rinsed with 2 changes of tap water and left to dry. Images of the colonies were taken using the Chemidoc Imaging System (Bio‐Rad) under the Coomassie Blue Stain filter setting. The total area and the number of colonies were estimated using the ImageJ Analyze particle option using 3 to 3500‐pixel units with a circularity of 0.2–1.

### 6.14 Immunofluorescence Staining and Confocal Imaging

Spheroids were fixed in 4% PFA for 1 h. After three washes with PBS, spheroids were stained with BV711 Rat Anti‐Mouse CD45 antibody (1:200, BD Biosciences) in PBS containing 3% BSA and 0.01% Tween‐20 for three days at 4°C in the dark. Spheroids were washed three times in PBS for 15 min. Spheroids were then mounted on glass slides with mounting media (VECTASHIELD Vibrance). Slides were allowed to dry overnight at room temperature before imaging. Z‐stacks of the spheroids were imaged with the Olympus FV3000 Confocal Microscope with 10x/0.40 objective lenses using diode lasers 488 nm (CFSE) and 640 nm (CD45). Images were analyzed using FV31‐ASW software and ImageJ.

### 6.15 Mouse Model and Imaging

All animal procedures (R25i‐0126) were approved and conducted in accordance with the Institutional Animal Care and Use Committee (IACUC, NUS) guidelines. Ten‐week‐old female BALB/c mice were acclimatized and randomized into sham (control) and PEMF (treatment) groups. To establish the tumor model, 1 × 10^5^ 4T1‐12b cells were injected subcutaneously into the dorsal flanks. 5 days after tumor inoculation, baseline tumor sizes were measured by in vivo imaging (IVIS). Longitudinal tumor progression was monitored every 7 days using the Xenogen IVIS Spectrum Imaging System (PerkinElmer). Briefly, mice received a subcutaneous injection of 100 μL of Luciferin (150 mg/kg) (MedChemExpress) 10 min prior to imaging. Tumor burden was quantified by defining regions of interest (ROIs) over the bioluminescent signal, and total flux (photons/sec) was measured using Living Image software (PerkinElmer).

### 6.16 PEMF Device

The PEMF device used for in vitro studies and its specifications were as previously described [15]. Briefly, the device produces spatially homogeneous, time‐varying magnetic fields consisting of barrages of 20 × 150 μs on‐and‐off pulses, repeated at 15 Hz for 6 ms. The magnetic flux density rose to a predetermined maximal level within ∼50 μs (∼17 T/s) when driving field amplitudes between 0.5 and 3 mT. Unless explicitly noted, all samples were exposed once for 10 min at ambient room temperature. Given the brief exposure duration, the media pH was not significantly affected by ambient temperature exposure. All PEMF‐treated samples were compared to time‐matched control samples (0 mT) that were manipulated identically as experimental samples, including placement into the PEMF‐generating apparatus for the designated time, except that the apparatus was not set to generate a magnetic field.

The PEMF device used in mouse studies was previously employed in a Phase 1 human clinical trial (ClinicalTrials.gov ID: NCT06332508), as well as in a published breast cancer mouse model [21]. Briefly, mice were placed in glass beakers positioned at the center of the PEMF device for 30‐min exposures twice a week. The entire mouse was exposed to the coil system. Control mice were treated identically, except that the device was set not to generate magnetic fields. Mice were not sedated or anesthetized during exposure and were returned to their cages immediately after treatment.

### 6.17 Statistical Analysis

All statistical analyses were performed using Graphpad Prism Version 10.5.0 software. Comparisons between two independent samples were analyzed using Student's t‐test. Comparisons between more than two independent samples were analyzed using one‐way analysis of variance (ANOVA) followed by Šidák's multiple comparison post hoc test. Comparisons between samples with more than one variable (e.g., time‐point and magnetic field exposure) were analyzed using two‐way ANOVA followed by Šidák's multiple comparison post hoc test. *p*‐value < 0.05 was considered statistically significant.

## Author Contributions

**Viresh Krishnan Sukumar:** methodology, validation, formal analysis, investigation, data curation, writing – original draft, visualization, project administration. **Yee Kit Tai:** conceptualization, methodology, writing – original draft, visualization, supervision, project administration. **Jan Nikolas Iversen:** investigation, validation. **Olivia Yeo:** investigation, validation. **Anisha Praiselin Paul:** investigation, validation. **Kwan Yu Wu:** visualization, investigation, validation. **Lina Hsiu Kim Lim:** conceptualization, resources, writing – review and editing, supervision. **Alfredo Franco‐Obregón:** conceptualization, resources, writing – review and editing, supervision, funding acquisition.

## Ethics Statement

All experiments involving animals were conducted in accordance with the ethical policies and procedures approved by the Institutional Animal Care and Use Committee (IACUC, NUS) (R22‐0962) and (R25i‐0126) for studies involving animals. Generative AI was used solely to improve the language clarity of the manuscript. All authors reviewed and edited the content as needed.

## Conflicts of Interest

A.F.‐O. is an inventor on patent WO 2019/17863 A1, System and Method for Applying Pulsed Electromagnetic Fields and a founder of QuantumTx Pte. Ltd., which elaborates muscle‐targeted electromagnetic field devices for human use. A.F.‐O. did not participate in the generation or actual analyses of the data. All other authors declare no conflicts of interest.

## Supporting information


Supporting Information S1

## Data Availability

All data are available in the main text or the supporting information.
