# Supplementary material for: Magnetic Reprogramming of Macrophages Stimulates Phagocytosis of Breast Cancer Cells via a TRPC1‐STING Inflammatory Axis
Source: Smart Med. 2026 Jun 4;5(3):e70038. doi: 10.1002/smmd.70038 (PMC13431815; doi:10.1002/smmd.70038)
Supplement: Supplementary file 1 — Supporting Information S1 [file SMMD-5-e70038-s001.docx]

Supporting Information

Magnetic Reprogramming of Macrophages Stimulates Phagocytosis of Breast Cancer Cells via a TRPC1-STING Inflammatory Axis

Viresh Krishnan Sukumar, Yee Kit Tai*, Jan Nikolas Iversen, Olivia Yeo, Anisha Praiselin Paul, Kwan Yu Wu, Lina Hsiu Kim Lim*, Alfredo Franco-Obregón*


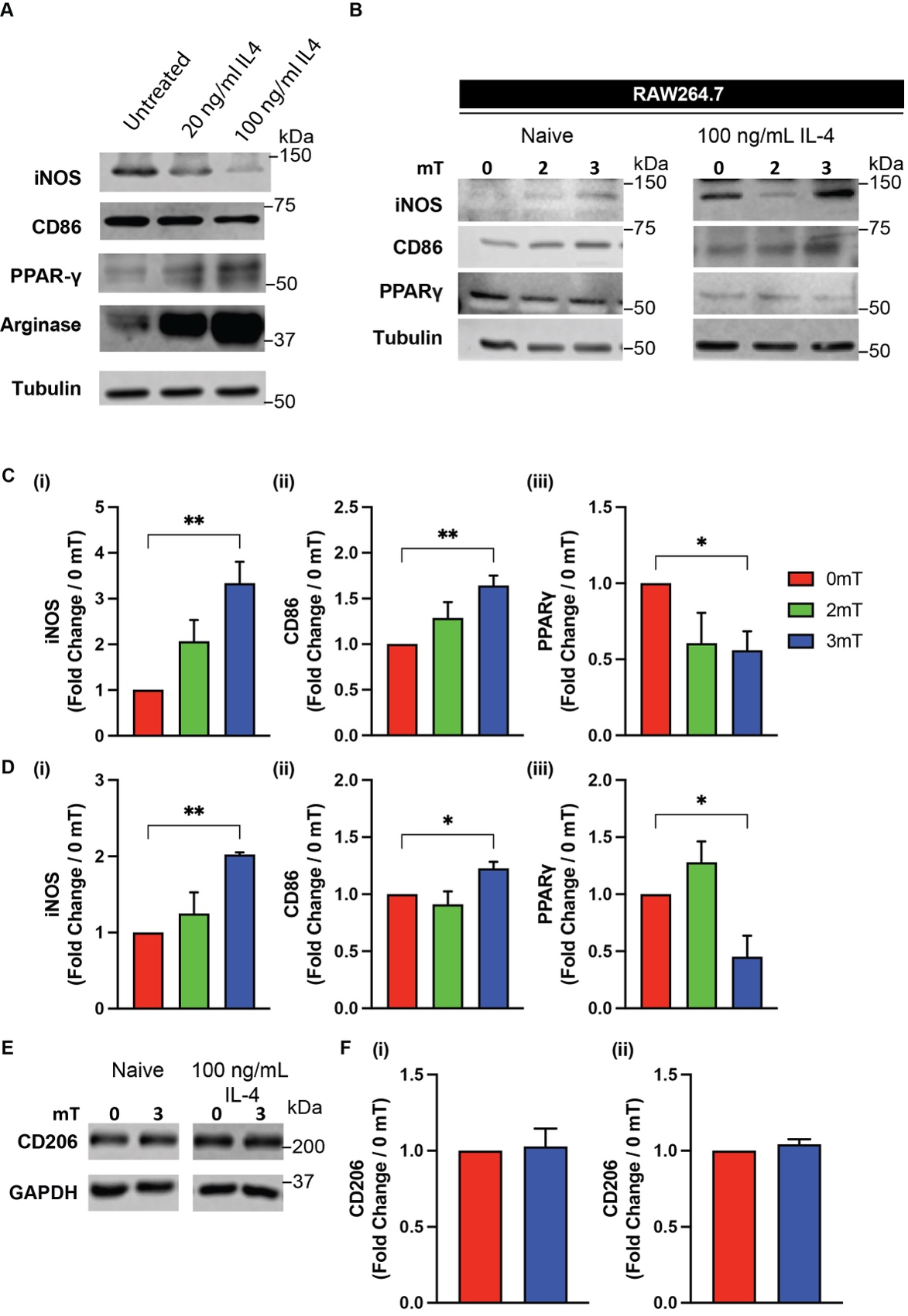


**Figure S1. PEMF (3 mT, 10 min) induces sustained (48 h) changes in macrophage polarization markers.** (A) Representative blots of validation of effects of IL-4 treatment on polarization markers. Representative western blot for macrophage polarization markers in PEMF-exposed (B) naïve and IL-4 (100 ng/mL, 24 h) treated RAW264.7 48 h after PEMF exposure. (C) Fold change quantification of (i) iNOS, (ii) CD86, (iii) PPAR-γ in PEMF-exposed RAW264.7 naïve macrophages and (D) Fold change quantification of (i) iNOS, (ii) CD86, (iii) PPAR-γ in PEMF-exposed IL-4 treated RAW264.7 macrophages. (E) Representative western blots and (F) fold change quantification of CD206 in (i) naïve and (ii) IL-4 treated RAW264.7 macrophages 24 h after PEMF exposure. Data represent mean ± standard error of the mean (SEM) (n = 3) and were analyzed using one-way ANOVA, followed by Dunnett’s multiple comparison post hoc test. Statistical significance is indicated by *, p ≤ 0.05 and **, p ≤ 0.01.


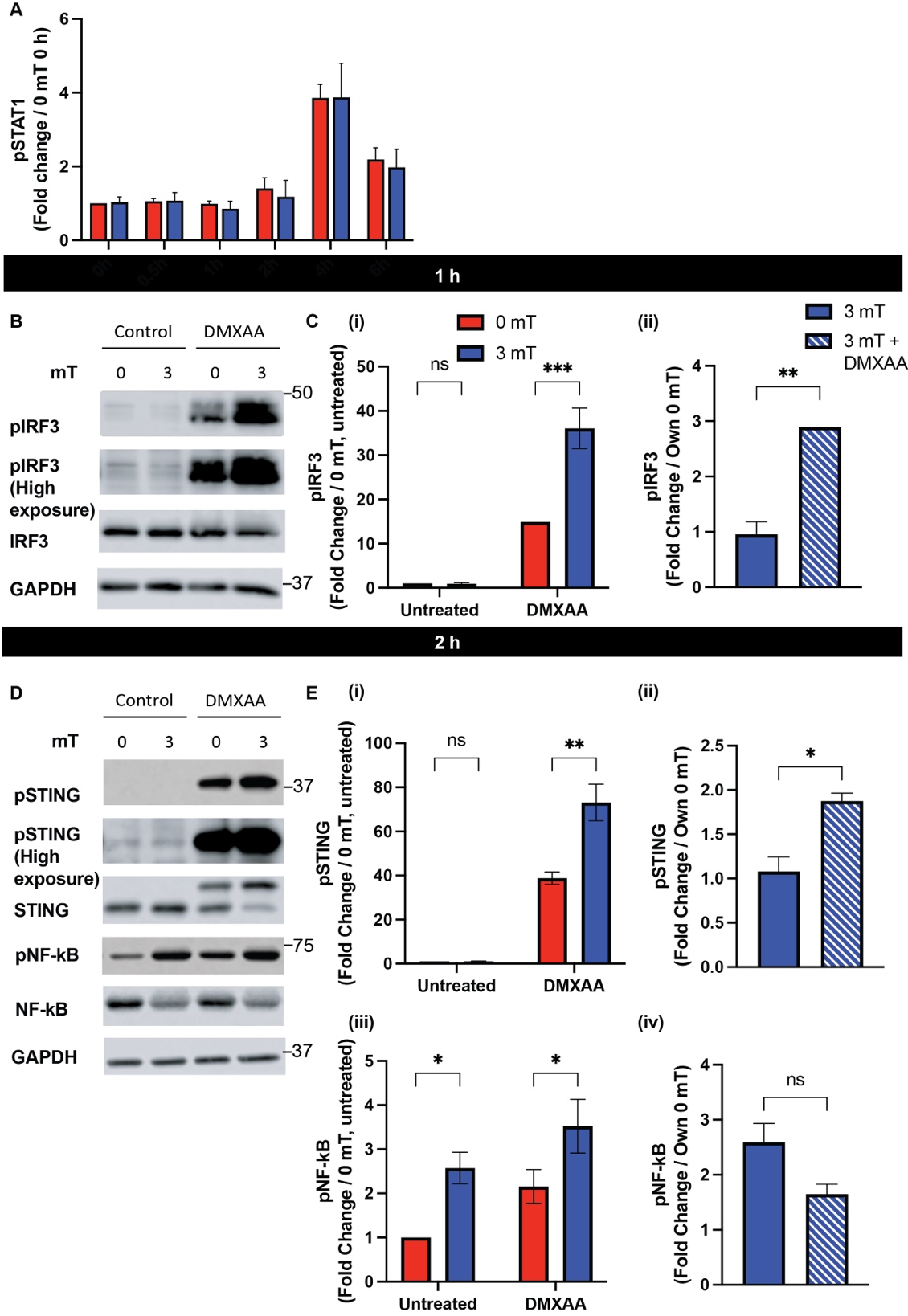


**Figure S2.** (A) Fold change quantification of phosphorylated STAT1 in RAW264.7 macrophages exposed to PEMF and STING agonist DMXAA (20 µg/mL) in a time course study. (B) Representative western blots and (C) fold change quantification of pIRF3 normalized to (i) 0 mT, no DMXAA or (ii) to each sample’s respective 0 mT control 1 h after DMXAA treatment. (D) Representative western blots and (E) fold change quantification of pSTING normalized to (i) 0 mT, no DMXAA or (ii) to each sample’s respective 0 mT control and pNF-kB normalized to (iii) 0 mT, no DMXAA or (iv) to each sample’s respective 0 mT control 2 h after DMXAA treatment. Phosphoprotein levels were normalized to their respective total protein abundance. Data represent mean ± standard error of the mean (SEM) (n = 3) and were analyzed using 2-way ANOVA, followed by Šidák’s multiple comparison post hoc test (**p*≤0.05, ***p*≤0.01, ****p*≤0.001 and ****, *p* ≤ 0.0001).


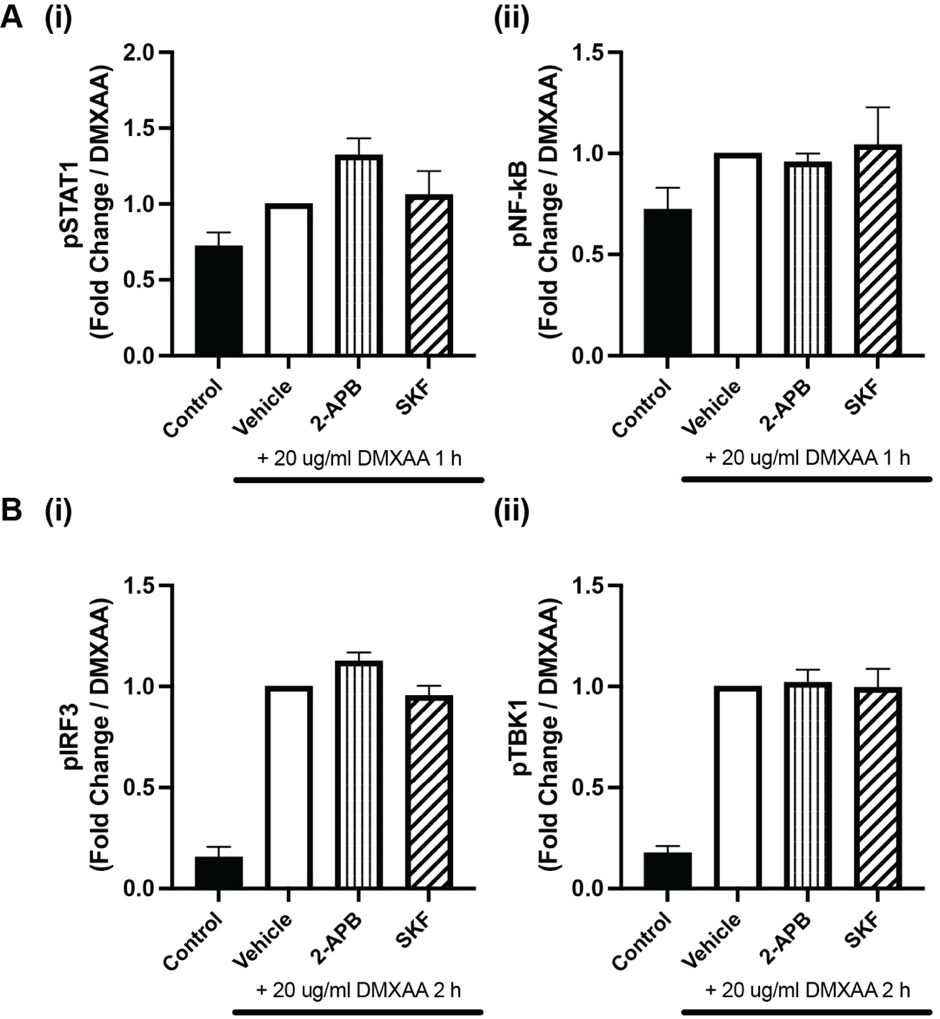


**Figure S3**. (A) Fold change quantification of phosphorylated (i) STAT1 and (ii) NF-KB 1 h after DMXAA treatment and phosphorylated (B) (i) IRF3 and (ii) TBK1 2 h after 20 µg/mL DMXAA treatment in RAW264.7 macrophages. Phosphoprotein levels were normalized to their respective total protein abundance. Data represent mean ± standard error of the mean (SEM) (n = 3) and were analyzed using one-way ANOVA, followed by Šidák’s multiple comparison post hoc test.


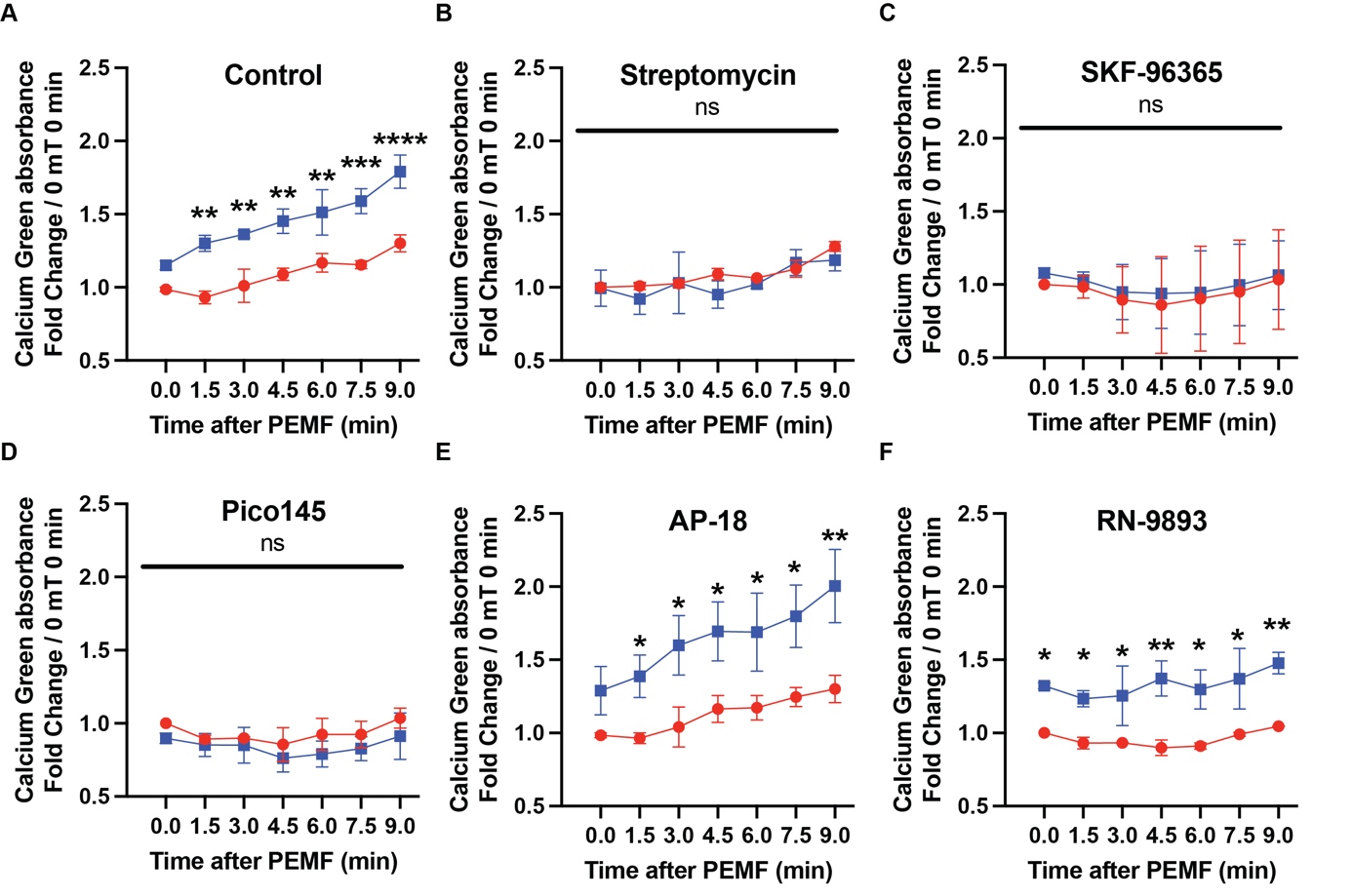


**Figure S4.** **Calcium Imaging in 4T1 cells with Calcium Green immediately after PEMF exposure with TRP channel inhibitors** (A) Control, (B) Streptomycin, (C) SKF-96365, (D) Pico145, (E) AP-18, (F) RN-9893. Data represent mean ± standard error of the mean (SEM) (n = 3) and were analyzed using 2-way ANOVA, followed by Šidák’s multiple comparison post hoc test (**p*≤0.05, ***p*≤0.01, ****p*≤0.001 and ****, *p* ≤ 0.0001).


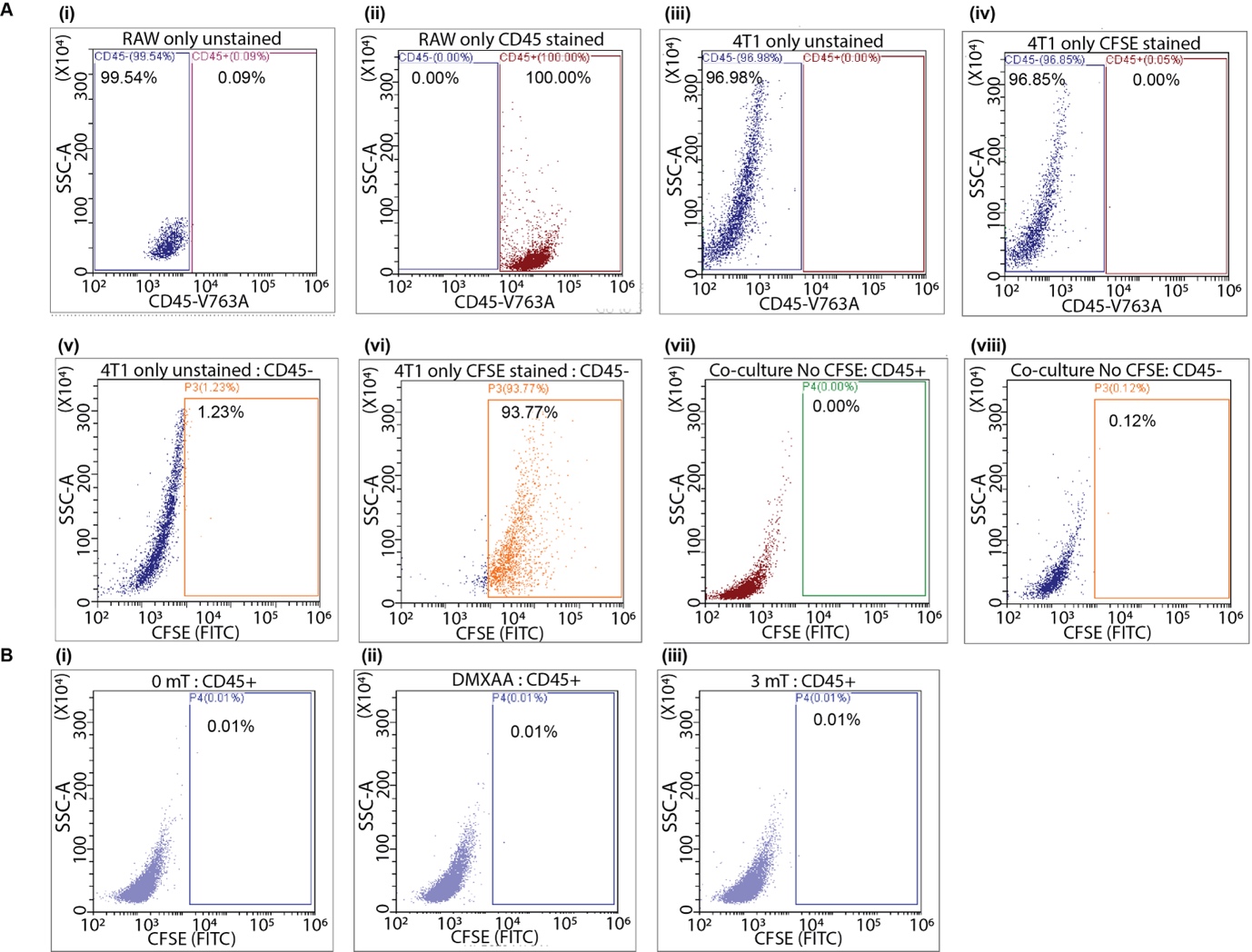


**Figure S5**: (A) Single-stained and unstained controls for FACS and phagocytosis experiments. (B) CFSE-stained 4T1 co-cultured with RAW264.7 with 0.4µm co-culture insert subjected to phagocytosis assay. (i) 0 mT, (ii) DMXAA, (iii) 3 mT (n=2).

**
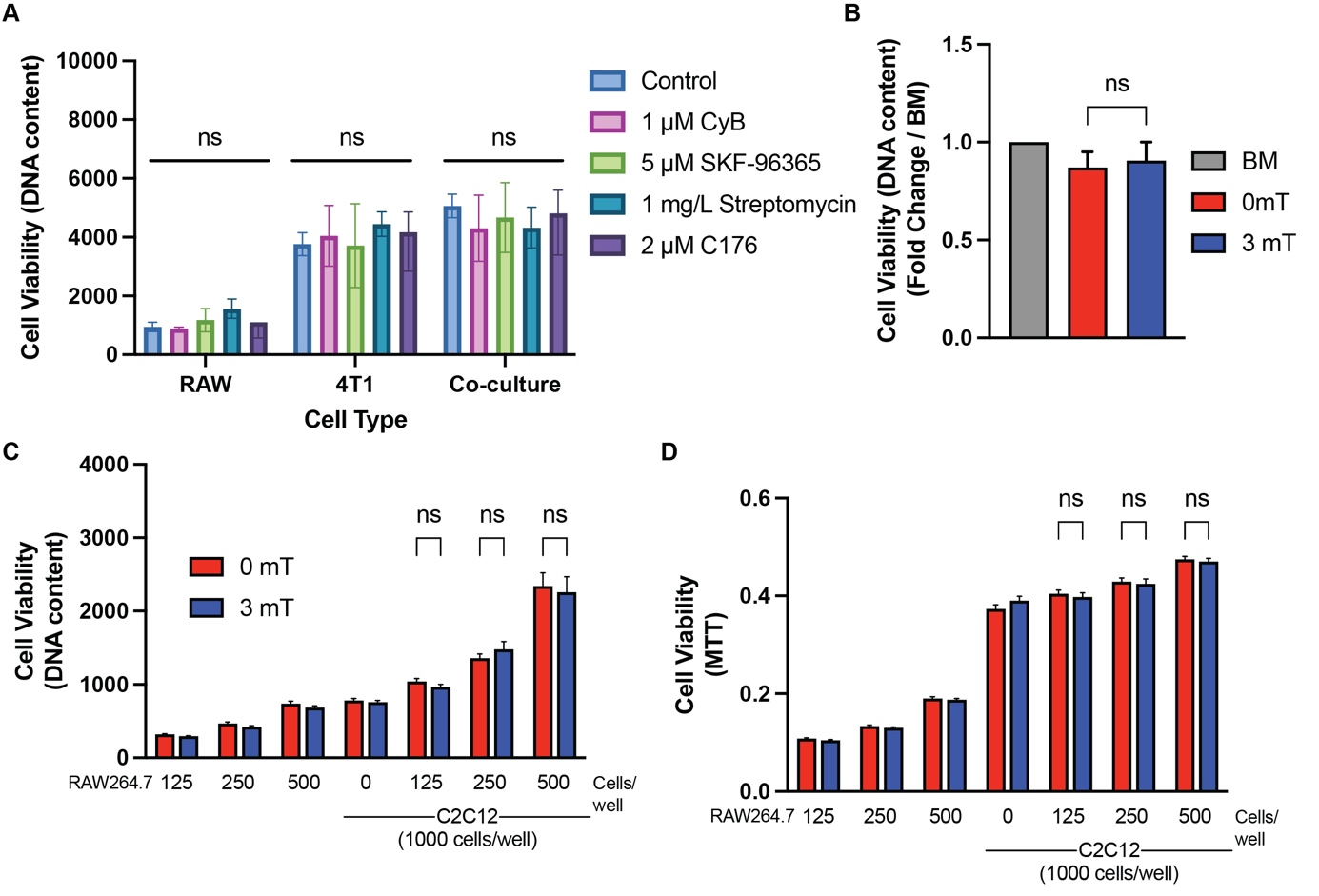
 Figure S6:** (A) Cell viability measured by DNA content (CyQuant) of RAW264.7, 4T1 or co-culture exposed to phagocytosis (1 µM Cytochalasin B), TRPC1 (5 µM SKF-96365, Streptomycin) or STING (2 µM C176) inhibitors. (B) 4T1 cell viability in response to conditioned media from PEMF-exposed RAW264.7 macrophages normalized to 4T1 grown in basal media (BM). (C) Cell viability measured by DNA content (CyQuant) of C2C12 co-cultured with RAW264.7 macrophages and exposed to PEMF. (D) Cell viability measured by metabolic activity (MTT) of C2C12 co-cultured with RAW264.7 macrophages and exposed to PEMF. Data represent mean ± standard error of the mean (SEM) (n = 3) and were analyzed using 2-way ANOVA, followed by Šidák’s multiple comparison post hoc test.


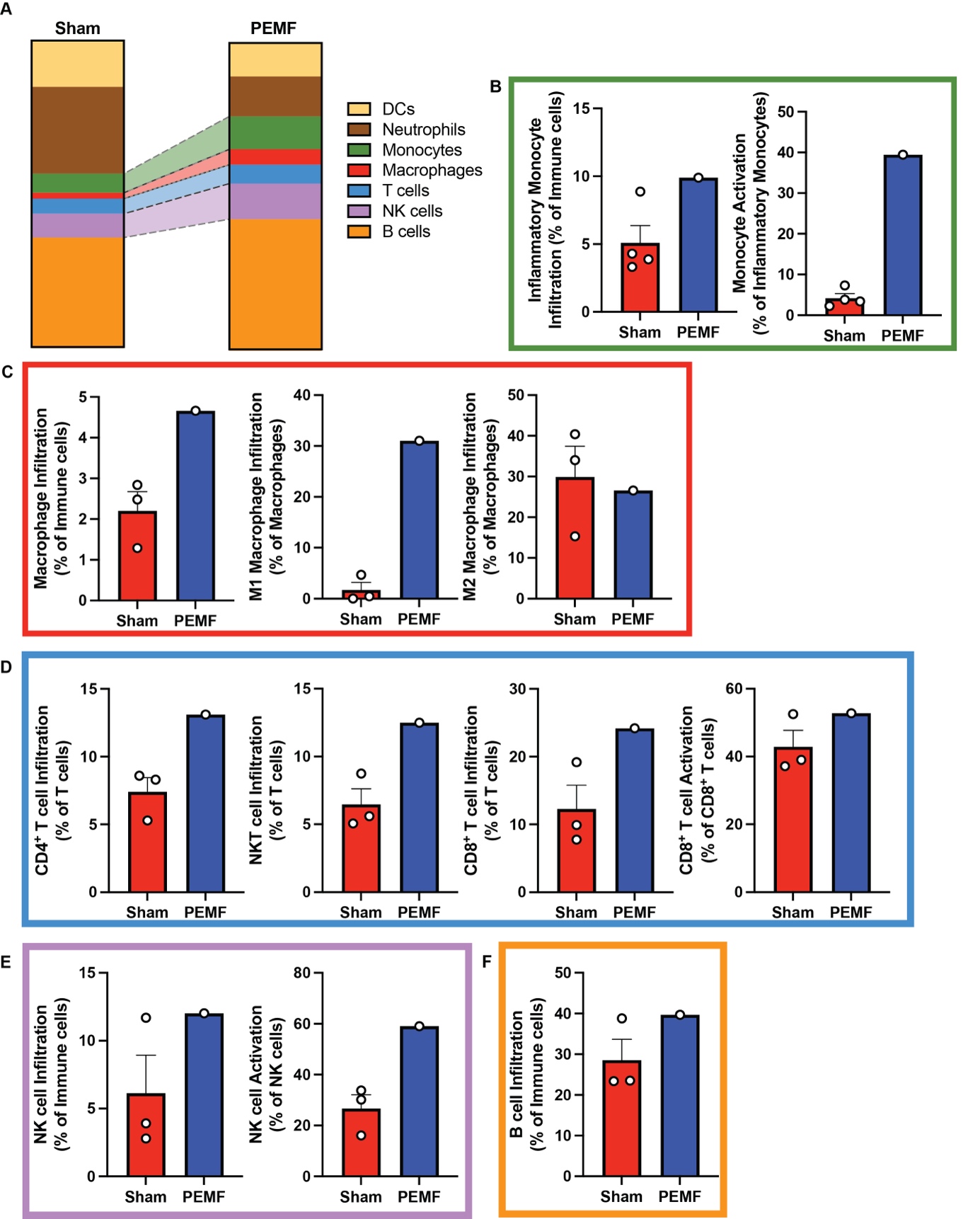


**Figure S7**: (A) Summary of immune profiling of 4T1 tumors. (B) Changes in Monocyte population (CD45^+^CD11b^+^Ly-6G^-^Ly-6C^+^, Activated – CD40^+^) (C) Changes in Macrophage population (CD45^+^CD11b^+^ Ly-6G^-^Ly-6C^-^MHCII^+^CD11c^-^, M1 – CD40^+^CD86^+^, M2 – CD206^+^). (D) Changes in T cell population (CD45^+^CD3^+^, CD8/CD4 for corresponding T cells, CD3^+^CD335^+^ for NKT cells, Activated - CD69^+^.) (E) Changes in NK cell population (CD45^+^CD3^-^CD335^+^, Activated - CD69^+^.) (F) Changes in B cell population. (CD45^+^ CD3^-^CD19^+^). Dots represent individual biological replicates. Data represents mean ± SEM.

**Table S1. dsiRNA sequences for knockdown of STING, STIM1 and TRPC1**

| **Target** | **dsiRNA Sequences** |
| --- | --- |
| STING dsiRNA 1 | 5' - GAAUCGGGUUUAUUCCAACAGCGTC -3'  3' - UUCUUAGCCCAAAUAAGGUUGUCGCAG -5' |
| STING dsiRNA 2 | 5' - CUUCUUAAUAAACAUAUCUAUUCTC -3'  3' - CGGAAGAAUUAUUUGUAUAGAUAAGAG -5' |
| STIM1 dsiRNA 1 | 5'- GAUGAGAUCAACCUUGCCAAGCAGG -3'  3'- CUCUACUCUAGUUGGAACGGUUCGUCC -5' |
| STIM1 dsiRNA 2 | 5' - AUGGUGAGGAUAAGCUUAUCAGCGT -3'  3'- GGUACCACUCCUAUUCGAAUAGUCGCA -5' |
| TRPC1 dsiRNA 1 | 5' - GCUGUUGAUAUACUACUUAAUCATC -3'  3'- CUCGACAACUAUAUGAUGAAUUAGUAG -5' |
| TRPC1 dsiRNA 2 | 5'- GUCCAGCUCUGAUAAUGUUAACAGA -3'  3'- GUCAGGUCGAGACUAUUACAAUUGUCU -5' |

Table S2. List of antibodies used for Western Blot analysis

| **Target** | **Brand** | **Cat no.** | **Dilution** |
| --- | --- | --- | --- |
| Phospho-Stat1 (Ser727) (D3B7) | Cell Signaling Technologies | 8826 | 1:1000 |
| Stat1 |  | 9172 | 1:1000 |
| Phospho-IRF-3 (Ser396) (4D4G) |  | 4947 | 1:500 |
| IRF-3 (D83B9) |  | 4302 | 1:1000 |
| Phospho-TBK1/NAK (Ser172) (D52C2) |  | 5483 | 1:500 |
| TBK1/NAK |  | 3013 | 1:1000 |
| Phospho-NF-κB p65 (Ser536) (93H1) |  | 3033 | 1:500 |
| Phospho-NF-κB p65 (Ser468) |  | 3039 | 1:1000 |
| NF-κB p65 (D14E12) |  | 8242 | 1:1000 |
| Phospho-STING (Ser365) (D8F4W) |  | 72971 | 1:500 |
| STING (D2P2F) |  | 13647 | 1:1000 |
| Phospho-IκBα (Ser32) (14D4) |  | 2859 | 1:1000 |
| IκBα (44D4) |  | 4812 | 1:1000 |
| Phospho-IKKα/β (Ser176/180) (16A6) |  | 2697 | 1:1000 |
| IKKβ (D30C6) |  | 8943 | 1:1000 |
| CD47 (E2V9V) |  | 36096 | 1:1000 |
| CD206 (E6T5J) |  | 24595 | 1:1000 |
| cGAS (D3O8O) |  | 31659 | 1:1000 |
| PPAR-γ (D69) |  | 2430 | 1:500 |
| iNOS | Genetex | GTX130246 | 1:500 |
| Arginase 1 |  | GTX109242 | 1:300 |
| CD86 | Invitrogen | MA5-35211 | 1:1000 |
| GAPDH | Proteintech | 10494-1-AP | 1:10000 |
| β-Actin |  | 60006-1-1g | 1:10000 |
| α-Tubulin |  | 66031-1-1g | 1:10000 |
| TRPC1 |  | 19482-1-AP | 1:1000 |

**Table S3: List of primers used for qPCR**

| **Target** | **Forward Primer (5’ – 3’)** | **Reverse primer (5’ – 3’)** |
| --- | --- | --- |
| TNF- α | AGCACAGAAAGCATGATCCG | CTGATGAGAGGGAGGCCATT |
| IL-23 | AGCTCTCTCGGAATCTCTGC | TGTCCTTGAGTCCTTGTGGG |
| CXCL10 | ATCATCCCTGCGAGCCTATC | TCTTGATGGTCTTAGATTCCGGA |
| CXCL11 | AGAGATCTCCAAAGCCCAGG | AGCTTTCTCGATCTCTGCCA |
| B2M | GATGTCAGATATGTCCTTCAGCA | TCACATGTCTCGATCCCAGT |

**Table S4: List of antibodies used for FACS analysis of tumor**

| **Marker** | **Clone** | **Fluorophore** | **Brand** | **Cat No** | **Panel** |
| --- | --- | --- | --- | --- | --- |
| CD45 | 30-F11 | AF700 | eBioscience | 56-0451-82 | Myeloid  (1:300) |
| MHC CLASS II | M5/114.15.2 | APC-eFLOUR 780 |  | 47-5321-80 |  |
| CD40 | 1C10 | PerCP-eFluor™ 710 |  | 46-0401-82 |  |
| CD11c | N418 | PE-Eflour 610 |  | 61-0114-82 |  |
| LY-6C | HK1.4 | BV711 | BioLegend | 128037 |  |
| LY-6G | 1A8 | PE |  | 127608 |  |
| CD11b | M1/70 | FITC |  | 101206 |  |
| CD86 | GL-1 | APC |  | 105011 |  |
| CD206 | C068C2 | PE/Cy7 |  | 141719 |  |
| CD45 | 30-F11 | BV711 | BD | 563709 | Lymphoid  (1:400) |
| CD8a | 53-6.7 | BV421 |  | 563898 |  |
| CD3e | 145-2C11 | BUV395 |  | 563565 |  |
| CD69 | H1.2F3 | PE/Cy7 | eBioscience | 25-0691-82 |  |
| CD4 | GK1.5 | AF488 |  | 53-0041-82 |  |
| CD19 | 1D3 | PE-Cyanine5 | Invitrogen | 15-0193-82 |  |
| Live/Dead |  | Near IR |  | L34980 |  |

**Table S5: Gating strategy for immune cell identification by FACS**

| **Immune cell type** | **Gating** | **Remarks** | **Panel** |
| --- | --- | --- | --- |
| Neutrophils | CD45^+^CD11b^+^Ly6G^+^ |  | Myeloid |
| Inflammatory Monocytes | CD45^+^CD11b^+^Ly6G^-^Ly6C^+^ |  |  |
| Activated Inflammatory Monocytes | CD40^+^ | From Inflammatory monocyte population |  |
| Dendritic Cells | CD45^+^CD11b^+^Ly6G^-^Ly6C^-^MHCII^+^CD11c^+^ |  |  |
| Macrophages | CD45^+^CD11b^+^Ly6G^-^Ly6C^-^MHCII^+^CD11c^-^ |  |  |
| M1 Macrophages | CD40^+^CD86^+^ | From macrophage population |  |
| M2 Macrophages | CD206^+^ |  |  |
| T cells | CD45^+^CD3^+^ |  | Lymphoid |
| CD8 T cells | CD8^+^ | From T cell population |  |
| CD4 T cells | CD4^+^ |  |  |
| NKT cells | CD335^+^ |  |  |
| NK Cells | CD45^+^CD3^-^CD335^+^ |  |  |
| B Cells | CD45^+^CD3^-^CD19^+^ |  |  |

**Table S6: Compensation Matrix for Myeloid Panel**

| **Autofl.** | **Channel** | **-B525-FITC%** | **-B690-PC5.5%** | **-Y585-PE%** | **-Y610-mCHERRY%** | **-Y763-PC7%** | **-R660-APC%** | **-R712-APCA700%** | **-R763-APCA750%** | **-V660%** | **-IR885%** |
| --- | --- | --- | --- | --- | --- | --- | --- | --- | --- | --- | --- |
| 1.60 | B525-FITC |  | 0.00 | 0.61 | 0.17 | 0.24 | 0.00 | 0.20 | 0.16 | 0.04 | 17.47 |
| 0.43 | B690-PC5.5 | 7.03 |  | 16.51 | 61.53 | 4.10 | 4.98 | 9.57 | 5.02 | 54.82 | 45.91 |
| 0.32 | Y585-PE | 0.27 | 0.00 |  | 54.09 | 2.37 | 0.13 | 0.55 | 2.36 | 4.09 | 5.67 |
| 0.04 | Y610-mCHERRY | 0.09 | 0.00 | 31.65 |  | 0.88 | 0.36 | 0.29 | 1.36 | 1.36 | 7.52 |
| 0.03 | Y763-PC7 | 1.67 | 8.31 | 2.13 | 7.36 |  | 8.61 | 17.70 | 56.26 | 5.23 | 5.30 |
| 0.09 | R660-APC | 0.00 | 0.21 | 0.00 | 0.05 | 0.03 |  | 0.46 | 33.11 | 0.97 | 7.03 |
| 0.09 | R712-APCA700 | 0.01 | 28.71 | 0.01 | 0.04 | 0.11 | 36.59 |  | 13.54 | 81.32 | 7.69 |
| 0.29 | R763-APCA750 | 0.07 | 12.04 | 0.00 | 0.04 | 6.67 | 23.79 | 69.49 |  | 40.00 | 6.66 |
| 0.61 | V660 | 0.05 | 10.37 | 0.27 | 0.71 | 0.16 | 1.63 | 5.35 | 0.72 |  | 4.03 |
| 0.14 | IR885 | 0.00 | 0.00 | 0.00 | 0.00 | 0.17 | 0.00 | 0.00 | 1.61 | 0.00 |  |

**Table S7: Compensation Matrix for Lymphoid Panel**

| **Autofl.** | **Channel** | **-B525-FITC%** | **-Y675-PC5%** | **-Y763-PC7%** | **-R763-APCA750%** | **-V450-PB%** | **-V660%** | **-UV405%** | **-IR885%** |
| --- | --- | --- | --- | --- | --- | --- | --- | --- | --- |
| 10.82 | B525-FITC |  | 0.06 | 0.00 | 0.00 | 0.00 | 0.00 | 0.00 | 49.49 |
| 1.08 | Y675-PC5 | 0.00 |  | 0.50 | 18.20 | 0.00 | 0.12 | 0.00 | 2.87 |
| 0.46 | Y763-PC7 | 0.00 | 48.22 |  | 194.98 | 0.00 | 1.54 | 0.00 | 2.33 |
| 9.09 | R763-APCA750 | 0.00 | 1.49 | 0.41 |  | 0.00 | 2.28 | 0.00 | 0.00 |
| 132.80 | V450-PB | 0.00 | 0.00 | 0.00 | 0.00 |  | 3.31 | 0.00 | 0.00 |
| 40.69 | V660 | 0.00 | 5.91 | 0.00 | 0.00 | 0.00 |  | 0.00 | 0.00 |
| 42.26 | UV405 | 0.00 | 0.00 | 0.00 | 0.00 | 8.98 | 0.00 |  | 0.00 |
| 0.25 | IR885 | 0.00 | 0.00 | 0.22 | 30.69 | 0.00 | 0.00 | 0.00 |  |
